# Discovery of Cyclic Peptide Inhibitors Targeted on TNFα-TNFR1 from Computational Design and Bioactivity Verification

**DOI:** 10.3390/molecules29215147

**Published:** 2024-10-31

**Authors:** Jiangnan Zhang, Huijian Zhao, Qianqian Zhou, Xiaoyue Yang, Haoran Qi, Yongxing Zhao, Longhua Yang

**Affiliations:** 1 Key Laboratory of Advanced Drug Preparation Technologies, Ministry of Education, School of Pharmaceutical Sciences, Zhengzhou University, Zhengzhou 450001, China; zhangjn1022@163.com (J.Z.); zhaohj5837@163.com (H.Z.); xixiruan@163.com (Q.Z.); yxyang0206@163.com (X.Y.); qhr12311@163.com (H.Q.); 2 Henan Key Laboratory of Nanomedicine for Targeting Diagnosis and Treatment, Zhengzhou 450001, China

**Keywords:** molecular dynamics simulation, computational drug design, cyclic peptide, TNFα, TNFR1

## Abstract

Activating tumor necrosis factor receptor 1 (TNFR1) with tumor necrosis factor alpha (TNFα) is one of the key pathological mechanisms resulting in the exacerbation of rheumatoid arthritis (RA) immune response. Despite various types of drugs being available for the treatment of RA, a series of shortcomings still limits their application. Therefore, developing novel peptide drugs that target TNFα-TNFR1 interaction is expected to expand therapeutic drug options. In this study, the detailed interaction mechanism between TNFα and TNFR1 was elucidated, based on which, a series of linear peptides were initially designed. To overcome its large conformational flexibility, two different head-to-tail cyclization strategies were adopted by adding a proline-glycine (GP) or cysteine-cysteine (CC) to form an amide or disulfide bond between the N-C terminal. The results indicate that two cyclic peptides, R1_CC4 and α_CC8, exhibit the strongest binding free energies. α_CC8 was selected for further optimization using virtual mutations through in vitro activity and toxicity experiments due to its optimal biological activity. The L16R mutant was screened, and its binding affinity to TNFR1 was validated using ELISA assays. This study designed a novel cyclic peptide structure with potential anti-inflammatory properties, possibly bringing an additional choice for the treatment of RA in the future.

## 1. Introduction

Rheumatoid arthritis (RA) is a chronic systemic autoimmune disease that can cause symptoms of pain, stiffness, swelling, and deformity in the joints and possibly result in joint destruction and loss of function [1,2]. Current studies indicate that the pro-inflammatory cascade mediated by inflammatory factor disorders can cause RA, which is closely related to tumor necrosis factor α (TNFα) and tumor necrosis factor receptor 1 (TNFR1) [3].

**Figure 1:**
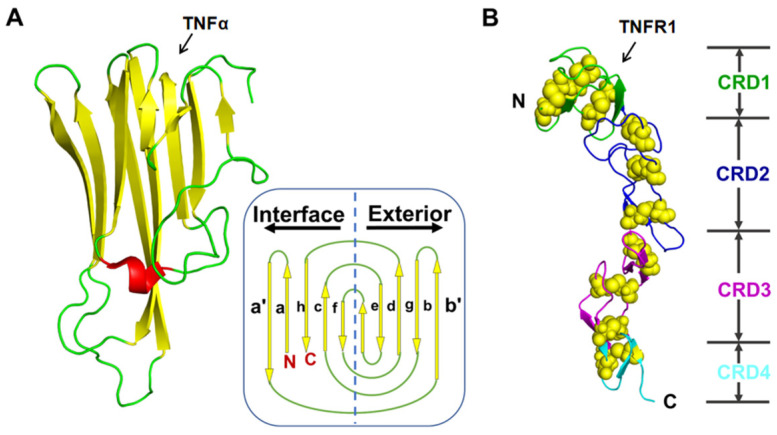
(**A**) The schematic diagram of the tertiary structure of TNFα, green and yellow represented the loop and β-sheet regions respectively. The lowercase letters in subfigure represent different β-sheets [9]. (**B**) The extracellular tertiary structure of TNFR1, cysteine, is represented by a yellow sphere. The four colors from top to bottom represent different regions of CRD1-4 (green, navy, purple, sky-blue). Both crystal structures were from the PDB bank (PDB ID: 7KPB [10,11]).

Structurally, TNFα belongs to type II transmembrane protein and is processed into soluble TNFα by a matrix metalloproteinase called TNFα converting enzyme (TACE), with a molecular weight of 17 kDa [4]. Specially, TNFα subunit represents a “jelly-roll” β-sheet sandwich structure, which is constructed almost entirely by antiparallel β-strands (Figure 1A) [5]. In vivo, biologically active TNFα molecule exits as a homotrimer, and activates the downstream signal pathway by binding to trimerized TNFα receptors (TNFR) on the cell surface, leading to receptor trimerization and activation, which in turn triggers a cascade of events leading to inflammation. In contrast, the TNFR1 receptor is a single-spanning type I transmembrane protein which shares the common structural features of most members of the TNF receptor superfamily members, which consists of two to four cysteine-rich domains (CRD1 to CRD4, from N-terminus to C-terminus) (Figure 1B) [6]. CRD1 (also known as PLAD) is essential for forming the TNFR self-complex on the cell surface [7], and the function of CRD4 remains unclear. CRD2 and CRD3, also known as TNF-binding domains, are the main interaction regions when TNFα binds to the TNFR1 receptor, which is crucial for drug design targeted on the protein–protein interaction between TNFα and TNFR1 [8].

As an important pleiotropic cytokine, TNFα has significant functions in both homeostasis and disease pathogenesis, with potent pro-inflammatory and immunomodulatory effects. TNFα can exert its activities by stimulating two different types of receptors expressed by macrophages, TNFR1 and TNFR2 [12]. TNFα binding to TNFR1 is primarily responsible for pro-inflammatory pathways, which play a dominant role in the pathogenesis and exacerbation of inflammation. The activation of TNFR1 can trigger the formation of different signaling complexes, resulting in distinct cellular responses [13]. During the assembly of complex I, the activated TNFR1 binds to TNFR1-associated death domain protein (TRADD), followed by the association and interaction of various components, including receptor-interacting serine/threonine-protein kinase 1 (RIPK1), TNFR-associated factor 2 or 5 (TRAF2/5), a cellular inhibitor of apoptosis protein 1 or 2 (cIAP1/2). This signaling pathway results in the activation of nuclear factor κB (NF-κB) and mitogen-activated protein kinases (MAPKs) [14,15,16]. Therefore, blocking the binding between TNFα and TNFR1 would be a promising strategy for the treatment of RA as this would inhibit the signal transduction of TNFα.

Currently, as one of the main drug types of TNFα inhibitors in the clinic, anti-TNF biologics, such as Infliximab, Etanercept, Adalimumab, Certolizumab, and Golimumab, have proven successful [17,18,19]. However, though this type is one of the best-selling classes of biologics, they have unwanted side effects, such as a high risk of infection, high cost, and induction of hematological malignancies [20]. Thus, developing other different types of TNFα inhibitors with fewer side effects, such as small molecules, aptamers, and peptides, is extremely urgent.

Several potential small molecules of TNFα inhibitors have already been reported. In 2005, He, M.M. et al. identified the first TNFα small molecule antagonist **SPD-304** (Figure S1A) with a K_d_ value of 5.36 μM. It could greatly increase the dissociation rate of the TNFα trimer, leading to the disassembly of the active form of the TNFα ligand [21]. Furthermore, through structure-based virtual screening and biological assay, Xie’s group identified two novel antagonists, **T1** and **R1** (Figure S1A), which could bind to TNFα or TNFR1, with low-micromolar binding affinities to TNFα and TNFR1, respectively [22].

In addition, Orava, E.W. et al. reported another type of TNFα inhibitor in 2013, the DNA aptamer **VR11** containing 25 bases (Figure S1B), which could recognize TNFα and inhibit TNFα signaling [23]. Moreover, our research group recently identified two aptamers with high affinity to TNFR1 through the Systematic Evolution of Ligands by Exponential Enrichment (SELEX), named **Apt1-67** and **Apt2-55** (Figure S1B), respectively [24]. Peptide drug development has made great progress in the last decade due to the advent of new production, modification, and analytical techniques [25].

Cyclic peptides offer several significant advantages over linear peptides, particularly in terms of stability, binding affinity, and bioavailability. The cyclic structure constrains the conformational flexibility of the peptide, enabling it to adopt a stable, predefined conformation that is ideal for binding to specific protein targets, such as TNFα-TNFR1 [26]. Cyclic peptide **WP9QY** (sequence: YCWSQYLCY), identified by Greene, M. I. et al., was shown to be able to disturb the TNFα-TNFR1 interaction through binding TNFα [27]. This triggered the development of protein–protein interactions (PPIs) inhibitors through the structure-based design of exocyclic peptidomimetic. Combining conformational analysis and molecular docking studies, in 2020, Houssen, W. E. et al. designed a novel cyclic peptide inhibitor (sequence: HYWSENLFQP) whose N-to-C-macrocyclic structure may provide better chemical stability and biological activity compared to disulfide-bonded peptidomimetic WP9QY [28]. However, none of these inhibitors, including small molecules, DNA aptamer, and peptide **WP9QY**, has yet entered clinical trials. Cyclic peptides are generally more resistant to proteolytic degradation compared to their linear counterparts, which is crucial for maintaining stability in biological environments such as blood circulation. This increased stability translates into a longer half-life, making cyclic peptides more suitable for therapeutic applications. More enhanced bioavailability allows them to penetrate cells more effectively, further supporting their potential as therapeutic agents. Thus, the cyclic peptides in this study were intentionally selected to maximize their therapeutic potential in targeting TNFα-TNFR1 [29].

As computer-aided approaches have become an integral part of the drug discovery process, identifying the exact interaction mechanism between TNFα and TNFR1 and performing mechanism-based drug design is a promising approach. In the present study, through the combination of theoretical calculation and bioassay validation, the interaction mechanism of TNFα and TNFR1 was explored in detail, and based on this, novel TNFα- and TNFR1-binding peptide inhibitors were designed and identified. The binding mode studies remind us to truncate a series of peptide fragments in the interaction first and then to improve the stability by different looping strategies. Then, the cyclic peptides with higher affinity were screened out by additional binding free energy calculations and in vitro cellular-level experiments assessing activity and toxicity. Finally, virtual mutations and ELISA experiments were performed to improve and verify the binding affinity. This work laid a solid theoretical foundation for understanding the TNFα-TNFR1 interaction mechanism in depth and also provided a new cyclic peptide inhibitor through a promising computational design strategy of the cyclic peptide for the treatment of inflammatory arthritis and autoimmune diseases.

## 2. Results and Discussion

### 2.1. TNFR1-TNFα Interaction Mechanism

#### 2.1.1. Structural Stability Analysis

**Figure 2:**
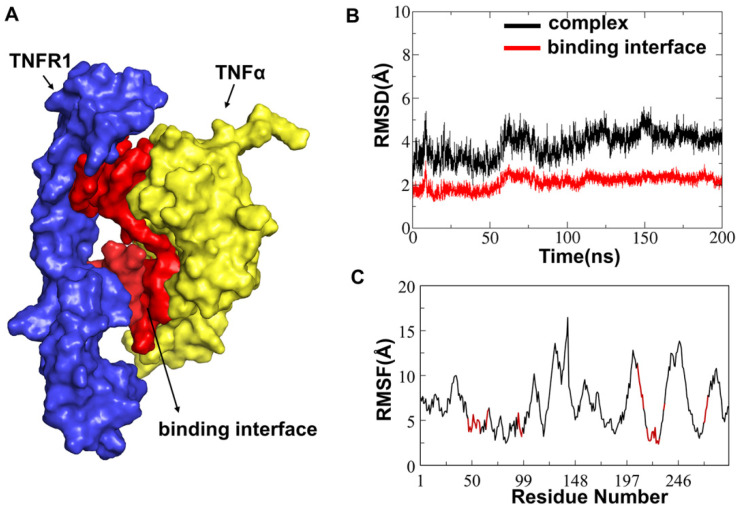
(**A**) Initial model of TNFα-TNFR1 complex (PDB ID: 7KPB). The interaction interface is shown in red. (**B**,**C**) RMSD and RMSF of the TNFα-TNFR1 complex (black) and the binding interface (red).

To evaluate the structural stability of TNFα-TNFR1 complex and its binding interface (Figure 2A), the root mean square deviation (RMSD) of heavy atoms was calculated for the residues in the entire system and for the residues within 5 Å of the binding interface. It was shown that the complex system reached equilibrium after 150 ns, with an average RMSD of 4.3 Å calculated by capturing the last 50 ns equilibrated trajectories (Figure 2B). As for the binding interface, the average RMSD was much smaller than that of the whole complex, only 2.1 Å, which represented greater stability (Figure 2B). This may be because the residues of the binding interface are mainly distributed in the CRD2-CRD3 region of TNFR1 and the β-sheet of TNFα, respectively, in structural. RMSF results (Figure 2C) indicated that during the whole simulation process, the interaction regions residues (Figure 2C marked as red) did not have apparent fluctuation, which indicated that the binding of TNFα to TNFR1 was stable as a whole, and the structure of the interaction regions was relatively conservative. This provided the prerequisite for the design of peptides.

#### 2.1.2. Identification of Key Residues in TNFα and TNFR1

##### Binding Free Energy and Free Energy Decomposition

In order to explore the binding mode between TNFR1 and TNFα, the binding free energy of the TNFα-TNFR1 complex was calculated using the MM-PBSA method based on the last 50 ns equilibrated trajectories. Table 1 lists the details of binding free energy and its components. The binding free energy between TNFα and TNFR1 was −50.80 kcal·mol^−1^, and the favorable contributions to binding free energy (∆G_bind_) came from the electrostatic energy (∆E_ele_), van der Waals interaction energy (∆E_vdw_) and the nonpolar solvation-free energy (∆G_nonp_).

**Figure 3:**
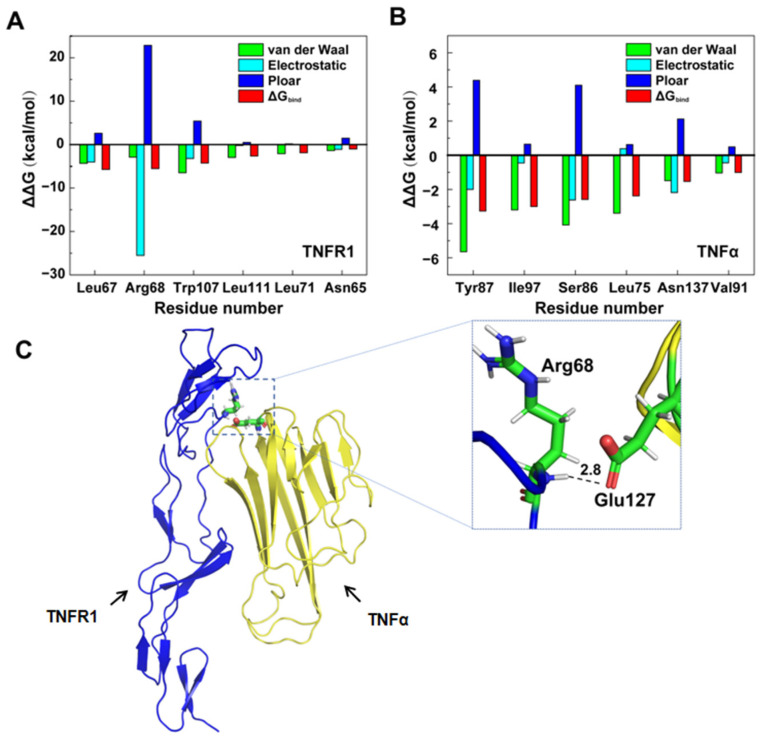
Per-residue free energy decomposition diagrams for TNFR1 (**A**) and TNFα (**B**). The residues with energy less than −1 kcal·mol^−1^ were plotted, and different energy terms are shown with different colors. (**C**) Hydrogen bond formed between Arg68 and Glu127.

We also calculated the per-residue binding free energy decomposition to explore the binding mode between TNFR1 and TNFα. Figure 3A,B show the top six residues contributed most to the interaction between TNFR1 and TNFα, respectively (per-residue binding free energy decomposition values all less than −1 kcal·mol^−1^). For TNFR1, except residue Arg68, the other top favorable contributors are all nonpolar residues with uncharged side chains. Hydrophobic residues Leu and Trp gave favorable contributions to the binding free energy mainly in the form of van der Waals and hydrophobic interactions. This was consistent with the binding free energy calculation results as well. Residue Arg68 was the second contributor after Leu67 (−5.74 kcal·mol^−1^), with the comparable binding free energy decomposition of 5.53 kcal·mol^−1^. Remarkably, due to the electropositivity, its electrostatic interaction contribution was much greater than the other residues, up to −25.51 kcal·mol^−1^. Combined with the hydrogen bond results (Table S1), it was found that the backbone N atom of Arg68 formed a strong hydrogen bond with the side-chain carboxylate oxygen of the electronegative Glu127 of TNFα, with the highest occupancy of 50.69% (Figure 3C and Table S2). Therefore, just like a “hook”, we identified this strong interaction as key for the interaction between TNFR1 and TNFα. As a result, we focused on this region and these important residues, ensuring they were retained in our truncated peptide designs.

As for TNFα, Tyr87 contributed the largest binding free energy, up to −5.65 kcal·mol^−1^ (Figure 3B and Table S3). Except for Asn137, the favorable contributions of van der Waals interactions to the binding energy were greater than the electrostatic interactions. Thereinto, Tyr87, Ser86, and Asn137 have comparable electrostatic interaction contributions, larger than −2.00 kcal·mol^−1^, which avoid their separation. Combined with the hydrogen bond results, Tyr87, Ser86, and Asn137 all have comparable electrostatic interaction contributions greater than −2.00 kcal·mol^−1^. Tyr87, Ser86 and Asn137 formed hydrogen bond with Ala62, His66, Trp107 of TNFR1, respectively (Table S2). It was concluded that van der Waals and hydrophobic interactions between uncharged residues are the main driving force for TNFR1 binding to TNFα. This may provide theoretical guidance for the subsequent design and optimization of inhibitors binding to TNFR1 or TNFα.

##### Computational Alanine Scanning

**Figure 4:**
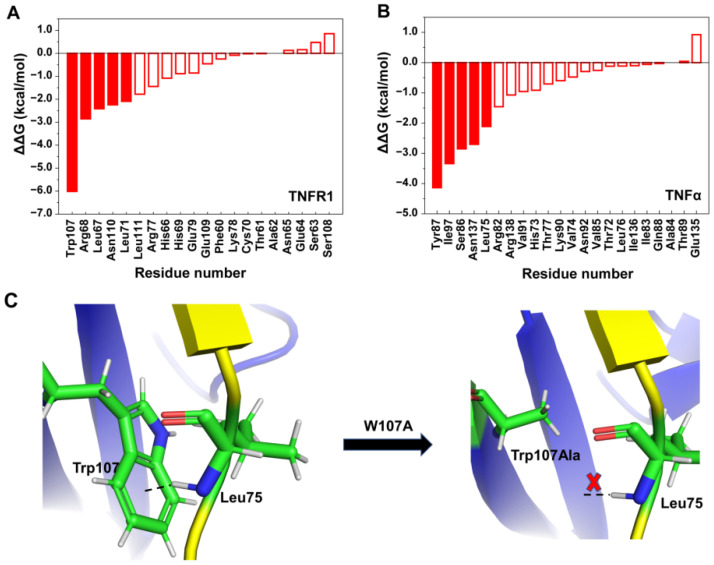
Binding free energy changes after mutations (within 5 Å of binding interface). (**A**) TNFR1; (**B**) TNFα. Hot-spot residues contributing −2 kcal·mol^−1^ are filled in red. (**C**) Interaction with Leu75 before and after Trp107 mutation. The blue and yellow ribbons represent TNFR1 and TNFα, respectively. X represents the interaction disappeared after mutation.

It is known that most protein–protein interactions are driven by a core region of the binding interface, and the residues in the core region are also called “hot-spot” residues [30]. In order to further confirm the important residues on TNFα and TNFR1, and to provide more detailed information about the binding mode, computational alanine scans were performed for the residues within 5 Å of the binding interface, and the changes of binding free energy (ΔΔG) between wild-type and mutants were calculated (Figure 4 and Table S4). As shown in Figure 4A, in TNFR1, there were five “hot-spot” residues (ΔΔG < −2 kcal·mol^−1^). In addition to the four residues (Leu67, Arg68, Leu71, and Trp107) that were consistent with the energy decomposition results, Asn110 also played an important role in the binding process. Furthermore, W107A occupied the largest binding free energy change due to the alteration of the hydrophobic side chain, which resulted in the removal of π-H interaction with Leu75 in TNFα (Figure 4C).

In TNFα, as shown in Figure 4B, there were also five “hot-spot” residues, including Leu75, Ser86, Tyr87, Ile97, and Asn137, which were identical to the important residues identified by binding.

**Figure 5:**
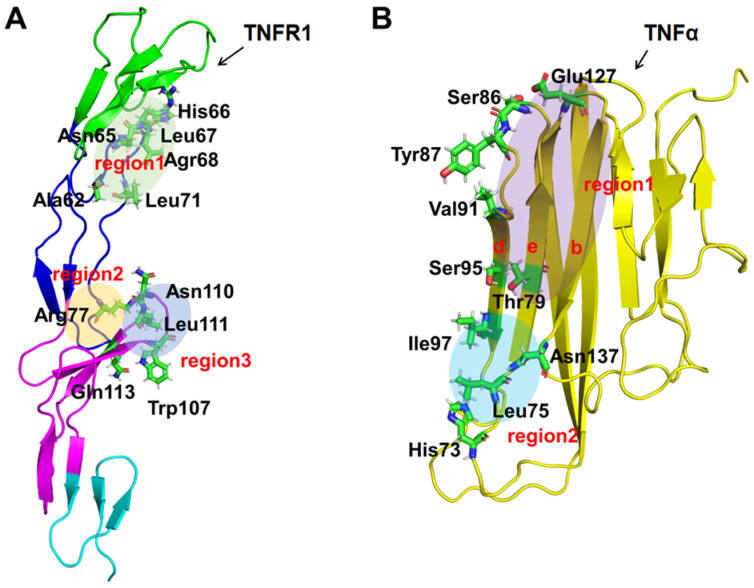
Binding sites schematic diagram of TNFR1 (**A**) and TNFα (**B**). The CRD1–4 regions of TNFR1 were represented from top to bottom.

Combining the results of binding free energy decomposition, hydrogen bond, and alanine mutation scans, the following conclusions were derived. There are three binding regions on TNFR1, which are mainly composed of loop1 and loop2 of CRD2, as well as loop1 of CRD3, while there are only two binding regions on TNFα, mainly located on its b, d, and e of β-sheets. There were 11 key residues on TNFR1 (Ala62, Asn65, His66, Leu67, Arg68, Leu71, Arg77, Trp107, Asn110, Leu111, and Gln113) and 10 key residues on TNFα (His73, Leu75, Thr79, Ser86, Tyr87, Val91, Ser95, Ile97, Glu127, and Asn137), respectively, which are essential for binding (Figure 5). These residues play significant roles in the binding of TNFα to TNFR1, which are critical for subsequent peptide truncation, selection, and design. In addition, these key residues were mostly uncharged, which focused our designs on including nonpolar interactions when designing corresponding antibodies and inhibitors to improve affinity.

### 2.2. Peptide Design

#### 2.2.1. Sequence Selection

Based on the explored interaction mechanism above, 11 peptide sequences were truncated, among which seven peptide sequences were from TNFR1, and four were from TNFα (Tables S5 and S6). When truncated, in order to ensure the high affinity and stability between the peptides and proteins, the sequence should be selected to contain as many key residues as possible. In this way, the maximum number of points of interaction was retained. Furthermore, the original tertiary structure of TNFα or TNFR1 was retained as intact as possible.

**Figure 6:**
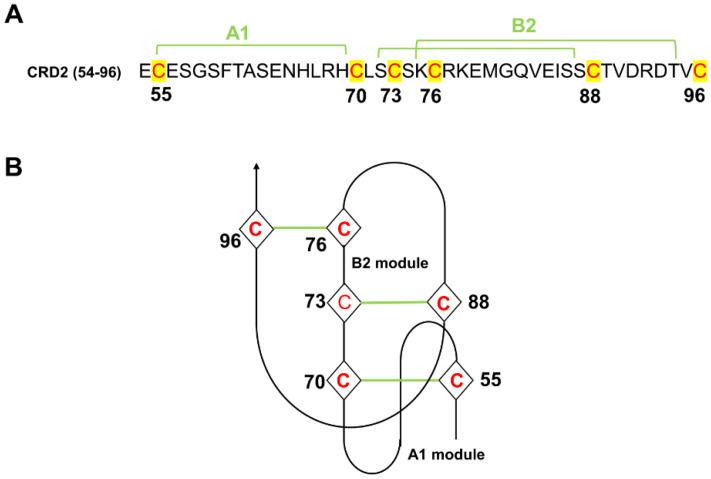
(**A**) Primary sequence and (**B**) topology of the CRD2 region in TNFR1.The green line represents the disulfide bond formed between two cysteines.

Following such thinking of the peptide truncation, for TNFR1 (Table 2), peptide **1** was obtained by truncating all key residues of Ala62-Leu71 in the core region 1. The sequences of peptide **2** and peptide **5** were obtained based on the two fragments that had been reported as the binding sites (Phe60–His66 and Cys76–Val83) between TNFR1 and TNFα by Greene, M. I. [27] On the basis of peptide **2** and **5**, peptide **3** and **6** (Table 2) were obtained by extending two residues on each terminal of peptide **2** and **5**, respectively, according to the similar residue extension method applied by Houssen, W. E. [28]. Three of the four cysteine-rich domains of TNFR1 are constructed from two distinct structural modules, termed A1 and B2 (Figure 6). Since both the A1 and B2 modules in the CRD2 region of TNFR1 were structurally conserved and stable, peptide **4** (Glu56–His69) and **7** (Ser84–Ser88) were obtained by retaining all residues of the A1 module and a portion of residues of the B2 module (Figure 6).

Using the similar principle of peptides truncation, four additional peptide sequences originating from TNFα were obtained. Among them, consecutive residues 79–95 were truncated as peptide **8**, since this truncation approach not only included all five key residues (Thr79, Ser86, Tyr87, Val91, and Ser95) already analyzed above but also preserved as many β-sheets as possible. The sequences of peptides **9**–**10** were all obtained based on the fragment reported by Greene, M. I. (Thr72–Leu77) and the key residues Ile97 and Asn137 analyzed above. Specifically, as shown in Figure 5, peptide **9** contained residues 72–77 and the adjacent residue Asn137 on the right; peptide **10** contained residues 72–77 and the adjacent residue Ile97 left in structural space; and peptide **11** contained both residues 72–77 and the two key individual residues (Ile97 and Asn137).

#### 2.2.2. Cyclization of Linear Peptides

However, the conformational flexibility of linear peptides always leads to a decrease in their binding stability and affinity. Taking the complex formed by linear peptide **2** and TNFα as an example, our simulations also confirmed the truncated linear peptide was subject to easy structural distortion, which would result in further separation from the target protein (Figure S2). Therefore, we also adopted the universal applied linear peptide cyclization method to overcome such disadvantages. Cyclization of linear peptides, either by backbone or side-chain linkage, can achieve much lower conformational flexibility and better drug-like properties. Furthermore, the rigidity of cyclic peptides decreases the entropy term of the Gibbs free energy, therefore allowing the enhanced binding toward target molecules or receptor selectivity [31,32,33].

Herein, two different head-to-tail cyclization strategies were adopted by adding a glycine-proline (GP) or cysteine-cysteine (CC) to form a disulfide or amide bond between the N-C terminal. So far, a total of 22 cyclic peptide structures have been obtained based on 11 linear peptide sequences applying these two cyclization strategies. According to the different original sources (TNFR1 or TNFα) and cyclization strategies (GP or CC), the peptides were named R1/α_GP/CCX. In the name, R1 or α identifies whether the sequence is from TNFR1 or TNFα, GP or CC identifies the cyclization strategy as proline-glycine or cysteine-cysteine, and X describes the peptide sequence serial number. Additionally, two cyclic peptides extracted from region 3 of TNFR1 reported in the literature [27,28] were chosen as the contrast and named R1_GP0 and R1_CC0, respectively.

#### 2.2.3. Peptide Screening

**Figure 7:**
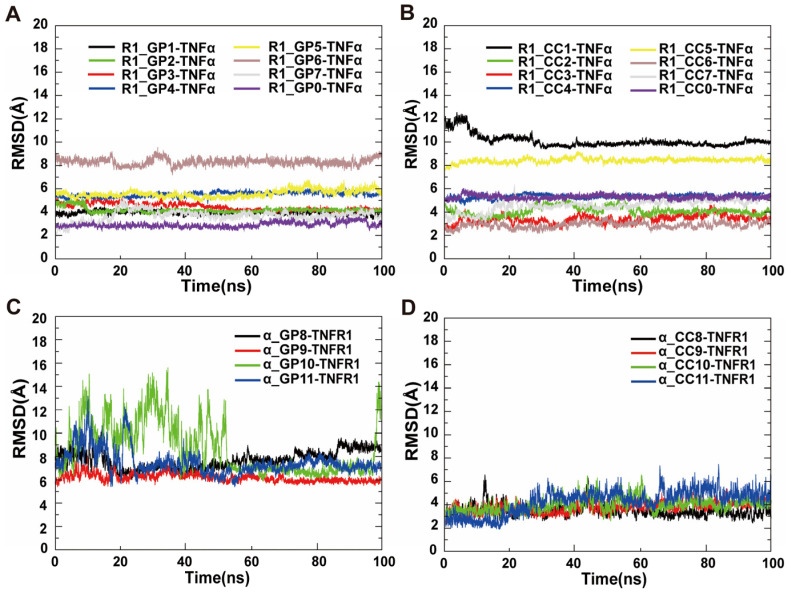
RMSD of different cyclic peptide-protein complexes. (**A**) RMSD of R1_GPX-TNFα complexes; (**B**) RMSD of R1_CCX-TNFα complexes; (**C**) RMSD of α_GPX-TNFR1 complexes; (**D**) RMSD of α_CCX-TNFR1 complexes.

In order to identify peptides with high affinity and stability subsequently, the corresponding cyclic peptide-protein complex initial models were constructed using HADDOCK (v2.4) software, and then the classical MD simulations and the MM-PBSA binding free energy calculations were performed. The RMSD curves of different CPs-TNFα (Figure 7A,B) and CPs-TNFR1 (Figure 7C,D) complexes were calculated based on the equilibrated 100 ns trajectory in each complex system. As shown in Figure 7A, for R1_GPX-TNFα complexes, the averaged RMSD values were in the range of 3–8 Å, and the overall fluctuations of the complexes were quite small, indicating that their binding was stable. For R1_CCX-TNFα complexes (Figure 7B), the averaged RMSD values were in the range of 3–10 Å, which was slightly higher than that of R1_GPX-TNFα complexes as a whole. Distinguishingly, for α_GPX-TNFR1 complexes (Figure 7C), RMSD values were around 8 Å, and the overall fluctuations were quite small except for α_GP10-TNFR1 complexes, which were excluded directly in the screening. However, this phenomenon did not appear in α_CCX-TNFR1 complexes. For α_CCX-TNFR1 complexes (Figure 7D), the RMSD fluctuations became smaller with the values of around 3 Å only.

Based on the equilibrated 50 ns trajectories, the binding free energies were calculated to further assess the strength of binding between the cyclic peptide and the target protein. For R1_GPX-TNFα complexes, R1_3GP had the strongest binding force to TNFα, with the binding free energy of −37.73 kcal·mol^−1^ (Table 2). For R1_CCX-TNFα complexes, R1_CC4 had the strongest binding force to TNFα, with the binding free energy of −41.13 kcal·mol^−1^, which was slightly higher than the strongest binding force to TNFα through amide bond cyclization strategy (α_GP3, −37.73 kcal·mol^−1^). Structurally, cyclized peptide structures maintain their cyclic shape without substantial structural alterations (Figure S4). Furthermore, it is speculated that the observed increase in binding affinity of R1_CC4 might be caused by the full retention of the A1 module in the CRD2 of TNFR1 region, which affords a more stable and rigid structure, resulting in stronger binding affinity.

Similarly, for the CPs-TNFR1 complexes (Table 3), peptide **8** had the strongest binding force to TNFR1, whether through disulfide bond cyclization or amide bond cyclization, with the binding energies of −23.98 kcal·mol^−1^ (α_GP8) and −35.10 kcal·mol^−1^ (α_CC8), respectively. Compared to the other peptides with less binding force, the α_CC8 fragment was a little longer, which can lead to the interaction more widely and imitate the β-sheet of TNFα better. Finally, R1_CC4 and α_CC8 were screened out for the subsequent biological activity verification and optimization.

#### 2.2.4. Bioactivity Measurement and Screening of the Cyclic Peptides

**Figure 8:**
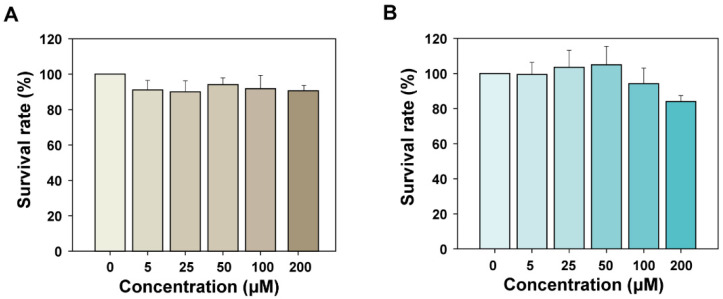
Cell toxicity of the different concentrations of cyclic peptides (**A**) cyclic peptide R1_CC4. (**B**) cyclic peptide α_CC8. Data represented as mean ± SEM (*n* = 3).

In this part, we screen the designed peptides through direct bioactivity experiments. The non-toxicity and safety of cyclic peptide inhibitors to normal cells are the basis of subsequent studies. A CCK-8 assay result showed that, except for 200 μM of α_CC8, the survival rate of L929 cells in the other group exceeds 90%, and there was no obvious inhibition of cell activity (Figure 8). This indicated that R1_CC4 and α_CC8 had no significant cytotoxicity to L929 cells. In addition, the cyclic peptide concentration was less than 200 μM in the subsequent evaluation of cell biological activity.

The mouse fibroblast cell line L929 is highly sensitive to TNFα-induced cell necrosis. Studies have shown that the TNFα-TNFR1 signaling pathway can lead to necrosis of L929 cells [34]. Therefore, the cytotoxic effect of TNFα on L929 cells can be evaluated by detecting the survival rate of L929 cells (Figure S3), and then the potential bioactivity ability of peptide R1_CC4 and α_CC8 can be verified by investigating the inhibition of R1_CC4 and α_CC8 on TNFα-mediated cytotoxicity.

**Figure 9:**
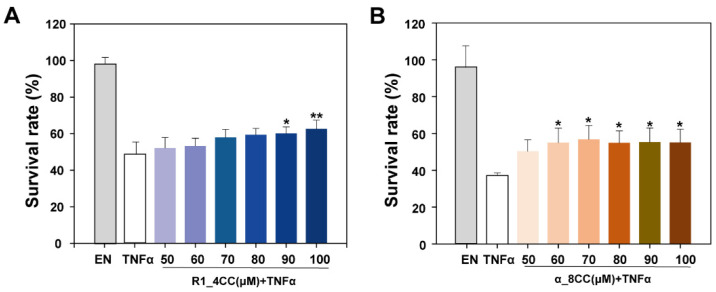
Survival rate of L929 cells in presence of 1 μg/mL Etanercept (EN positive control) and different concentrations of cyclic peptides. (**A**) cyclic peptide R1_CC4. (**B**) cyclic peptide α_CC8. Data are representative of three replicates. Data represented as mean ± SEM (*n* = 3). (* *p* < 0.05 and ** *p* < 0.01).

As shown in Figure 9, Etanercept, a TNFα inhibitor, was used as a positive control. After the incubation with TNFα for 17 h, the survival rate of cells was 42% on average, but the survival rate increased significantly after the incubation with cyclic peptides. When the concentration of cyclic peptide R1_CC4 and α_CC8 reached 90 μM and 60 μM, respectively, the survival rate increased significantly. The cell survival rate was increased by 11.6% and 17.8%, respectively. The results showed that both cyclic peptides had biological activity and could effectively inhibit the TNFα-mediated cytotoxicity. Etanercept is a fusion protein formed by the complete extracellular functional region of human TNFR2 and the Fc fragment of human IgG1, which has a strong ability to bind TNFα. This could explain why Etanercept had a stronger blocking effect on the formation of TNFα/TNFR1 and why the inhibitory effect of the peptide was lower than Etanercept. However, cyclic peptides can be modified or mutated easily; thus, there is still the possibility of improving both the affinity and stability further, compared to Etanercept. Because α_CC8 has better bioactivity and lower effective concentration than R1_CC4, it was used as the cyclic peptide template for the subsequent optimization and modification.

### 2.3. α_CC8 Based Optimization and Validation

#### 2.3.1. Optimization of α_CC8 Based on Mutation Strategy

In order to enhance the affinity of α_CC8, the interaction mechanism between α_CC8 and TNFR1 was identified by a series of computational simulation technologies, and then virtual mutations were performed based on the interaction mechanism. The PCA, FEL, and DCCM results demonstrated that α_CC8 could preserve the original β-sheet structure of TNFα to a significant degree and also partially overlapped the interaction sites between TNFα and TNFR1 (Figures S4–S6). This provided a guarantee for further mutations. As the binding free energy decomposition and hydrogen bond results revealed, electrostatic interaction was the main driving force of the binding, and a total of six important residues (Cys19, Asn15, Val14, Lys13, Thr2, and Tyr10) were identified as critical for good binding. These important residues were retained as key residues in subsequent virtual mutations (Tables S7–S10).

**Figure 10:**
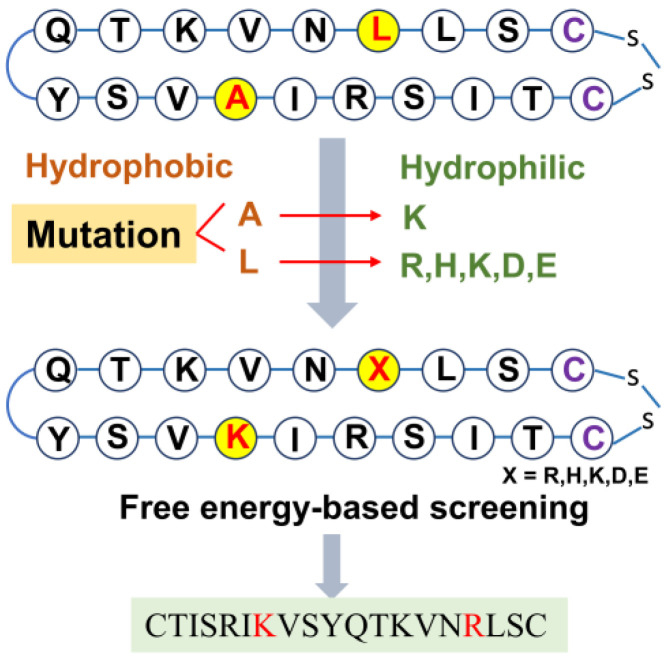
The virtual mutation flow chart of α_CC8.The red highlighted font represents the abbreviation of the amino acid to be mutated, the red arrow indicates the specific mutation process, and the red font at the bottom is the final screening result after mutation.

As shown in Figure 10, when performing virtual mutations, the design concept was to mutate the hydrophobic residues into charged residues to improve both affinity at TNFR1 and solubility. Additional solubility prediction, molecular dynamics simulations, and binding free energy calculations were subsequently performed. The results showed that among the five mutants, three mutants (L16R, L16H, L16K) had comparable binding free energy values with the original type (Table 4). Notably, the L16R mutant not only had higher solubility than the original type (Table S10) but also had a higher affinity with TNFR1 than the original type. Its binding free energy reached −39.03 kcal·mol^−1^ (Table 4 and Figure S7). Thus, L16R was considered an ideal mutant with high affinity and stability, which can be used for further analysis and research.

#### 2.3.2. Binding Affinity Verified by ELISA

To further measure the binding affinity of α_CC8 mutant peptide L16R with hTNF1, the ELISA experiments were performed, with the positive control of WP9QY, a reported but unvalidated cyclic peptide targeting TNFR1 [27].

**Figure 11:**
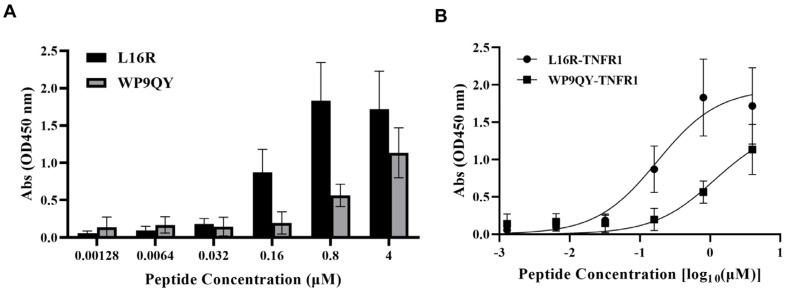
Binding affinities of L16R and WP9QY (positive control) toward the hTNF1, as determined by ELISA. (**A**) Absorbance values of L16R and WP9QY at different concentrations; (**B**) Binding affinity curves of cyclic peptide L16R and WP9QY. Data represented as mean ± SEM (*n* = 3).

Figure 11A shows the absorbance values measured at 450 nm under different cyclic peptide concentrations. Through analysis, we can find that the concentration of the two has a similar trend with the change of absorbance value and has a nonlinear dependence relationship. When the concentration was low, the absorbance values of the two groups did not change much, which was similar to that of the blank control group. When the concentration reached 0.16 μM, the absorbance values of the two groups began to change sharply. When the concentration reached 0.8 μM, the absorbance values of the two groups increased steadily and no longer changed. Figure 11B shows the changes in cyclic peptides of different concentrations with absorbance. The concentration was transposed to log_10_, and the binding affinity constant KD values of the two cyclic peptides were fitting [35]. The cyclic peptide L16R designed by us has a good binding condition with hTNFR1, KD value is 0.1768 μM, and the affinity constant fitting between WP9QY and hTNFR1 is 1.153 μM. It can be seen that the affinity of L16R is nearly 10 times higher than that of WP9QY. It is proved that our designed cyclic peptide has a good ability to target hTNFR1.

## 3. Materials and Methods

### 3.1. Computational Peptide Design

#### 3.1.1. Modeling Construction of TNFR1-TNFα/Cyclic Peptides Complexes

TNFR1 protein is a transmembrane protein of 455 residues, which contains a 29 amino acid N-terminal signal peptide, extracellular domain (residues 30–211), transmembrane (residues 212–232), and a C-terminal cytoplasmic domain (residues 233–455) that includes the death domain (residues 356–441) [8,36]. Therefore, the extracellular segment can bind to TNFα to cause an intracellular response, leading to the activation of downstream pathways. Therefore, we selected its extracellular region as the main research object. The initial TNFα-TNFR1 complex models were obtained from the crystal structure of TNFα-TNFR1 (PDB ID: 7KPB) [10]. Since in the TNFα and TNFR1 trimer, the TNF trimer has spatially distinct but equivalent receptor-binding sites [37], only a monomer of TNFα and TNFR1 (chain B and E) was retained for the purpose of model simplification. Residue repair, cyclization, and energy minimization were performed by Molecular Operating Environment (MOE) v2020 software [38]. The protonation states were titrated at pH 7.4 using H++ server [39].

Since HADDOCK’s (v2.4) performance is comparable to or better than the current state-of-the-art practice, especially for short cyclic peptides, it was used to conduct a series of dockings for the cyclic peptides (CPs) and TNFα or TNFR1 [40,41,42]. The hydrogen bond restraints were applied in its iteration, and the cutoff is 0.6 Å. The method for clustering is FCC. Based on this, 20 cyclic peptide-protein interactional models were built up through molecular docking between cyclic peptide and TNFR1 or TNFα.

#### 3.1.2. Molecular Dynamics Simulation

All MD simulations were carried out using the GPU-accelerated version [43,44] of the PMEMD program in the Amber 20 software package [45] and repeated three times. For the complex, the amber ff14SB force field parameters were applied [46]. TIP3P water model was applied for water in a cubic box, and the minimum distance between any solute atom and the edge of the box was 10 Å or 12 Å [47]. Sodium ions were added to neutralize the solvated system. Steepest descent and conjugate gradient methods were used for the subsequent energy minimization to optimize the structures of solvents and solutes. The temperature was slowly and smoothly heated to 300 K using the Langevin dynamic [48] with 5 ps^−1^ collision frequency. The equilibration was first performed at constant volume and then at constant pressure (1 bar) by the Berendsen barostat [49] with a pressure relaxation time of 2.0 ps. Production simulations were carried out at the NPT ensemble. The cutoff value for van der Waals (VDW) and short-range electrostatic interactions was set to 8.0 Å. Long-range electrostatic interactions were treated with the Particle-Mesh Ewald (PME) method. All covalent bonds involving hydrogen atoms were restricted with SHAKE algorithm [50]. In all simulations, the integral time step was 2 fs, and periodic boundary conditions were used. The simulation time of complexes was 100 ns and 200 ns, and equilibrated trajectories were analyzed using the AmberTools CPPTRAJ v17.0 program [51].

#### 3.1.3. Binding Free Energy Calculation

Considering a good balance between computational efficiency and accuracy, MM-PBSA (Molecular Mechanics Poisson–Boltzmann Surface Area) method [52] was chosen finally to calculate the binding free energy through the MMPBSA.py module of the Amber20 package [53]. MM-PBSA is a post-processing end-state method and is widely applied as an efficient and reliable free energy simulation method to model molecular recognition, such as cyclic peptide-protein binding interaction. When calculated, the last 50 ns trajectories were applied. The ionic strength was set to 0.15 M, and the external and internal dielectric constants were set to 80 and 1, respectively.

The binding free energy (ΔG_bind_) was computed by the following equations [54], where G_complex_, G_recepotor_, and G_ligand_ are free energies of complex, TNFR1, and TNFα or cyclic peptide, respectively.

(1) $${\Delta G}_{bind}={\Delta G}_{complex}-{\Delta G}_{receptor}-{\Delta G}_{ligand}$$

(2) $$\Delta G_{X}=\Delta H-T\Delta S\approx {\Delta E}_{MM}+\Delta G_{solv}-T\Delta S$$

(3) $$\Delta E_{MM}=\Delta E_{ele}+\Delta E_{vdw}+\Delta E_{int}$$

(4) $$\Delta G_{solv}={\Delta G}_{polar}+{\Delta G}_{nonpolar}$$

(5) $${\Delta G}_{nonp}=\gamma SASA+\beta$$

Here, T is the temperature, E_MM_ is gas phase molecular mechanical energy, G_solv_ is solvation-free energy, and E_ele_, E_vdw_ and E_int_ are electrostatic energy, van der Waals interaction energy and internal energy, respectively. In the study, the total value of E_int_ accounting for E_bond_, E_angle_, and E_dihedral_ (bond, angle, and dihedral energies) is zero. G_solv_ is calculated as the sum of polar contribution (G_PB_) to solvation-free energy calculated using the Poisson–Boltzmann (PB) model and nonpolar contribution (G_nonp_) calculated from a linear relation to solvent-accessible surface area (SASA). In Equation (5), two empirical constants, γ and β, were set as 0.00542 kcal·mol^−1^·Å^−2^ and 0.92 kcal·mol^−1^, respectively. The SASA was determined by a probe radius of 1.4 Å [55]. The change in conformational entropy −TΔS is usually calculated by normal-mode analysis on a set of conformational snapshots taken from MD simulations. However, because of the heavy computational cost, entropy is usually neglected.

#### 3.1.4. Alanine Mutation

To identify hot-spot residues that contributed binding free energy greater than −2 kcal·mol^−1^, residues within 5 Å of the TNFα-TNFR1 interaction interface were individually mutated to alanine. The alanine scanning method introduced mutations at a specific residue (x) by replacing it with alanine (a). Since the alanine side chain is composed of a simple alkyl group, it is assumed not to have a measurable contribution toward binding free energy values. Therefore, the contribution of the individual residue (x) could be calculated by the difference value before and after alanine mutations.

(6) $$∆∆G_{bind}^{x\to a}=∆G_{bind}^{x}-∆G_{bind}^{a}$$

where, $∆G_{bind}^{x}$ and $∆G_{bind}^{a}$ represent the binding free energy of the wild-type protein and alanine mutant, respectively [56]. The external and internal dielectric constants were set to 80 and 1, respectively.

### 3.2. Bioassay Validations

#### 3.2.1. Materials Preparation

L929 cell line was purchased from iCell Bioscience Inc. (Shanghai, China). Fetal Bovine Serum (FBS) was acquired from Biological Industries (Melbourne, Australia). RPMI Medium 1640 and Phosphate Buffered Saline (PBS) pH 7.4 were acquired from Solarbio (Beijing, China). Cell Counting KIT-8 (CCK-8) was acquired from meilunbio^®^ (Dalian, China). Dimethyl sulfoxide (DMSO) was purchased from Sigma-Aldrich Life Science & Tech. Co., Ltd., (Shanghai, China). Trypsin was acquired from Beyotime Biotechnology (Shanghai, China). Recombinant human TNFα was acquired from cusabio^®^ (Wuhan, China). Peptide R1_CC4 and peptide α_CC8 were acquired from Wuhan DGpeptides Co., Ltd., (Wuhan, China). Acetonitrile (HPLC/SPECTRO) was purchased from Tianjin Trunpu Weiye Technology Co., Ltd., (Tianjin, China). Synergy H1 full-function microplate reader was purchased from Bio Tek Instruments Co., Ltd., (Winooski, VT, USA). Thermo Forma 3111 CO_2_ incubator was purchased from Sheldon Company (Sheldon, ND, USA).

#### 3.2.2. Toxicity Properties of Peptide

Cytotoxicity of R1_CC4 and α_CC8 was assessed using the CCK-8 method. The final concentrations of cyclic peptide R1_CC4 and cyclic peptide α_CC8 were 0 μM, 5 μM, 25 μM, 50 μM, 100 μM, and 200 μM. L929 cells were seeded in a 96-well plate at a density of 8000 cells per well and cultured for 24 h under standard conditions. Then, the liquid in the wells was discarded, and 100 µL of medium containing different concentrations of cyclic peptides was added. After incubation for 24 h, the liquid in the well was discarded, and the medium was replaced with CCK-8 working solution (100 μL RPMI 1640 medium containing 5% CCK-8) and incubated in the dark for 2 h. The absorbance of each well at 450 nm was detected with a microplate reader. The same operation was performed with wells not seeded with cells as a blank group to eliminate systematic errors. Cell viability was calculated according to the following formula:

(7) $$Cell\;viability(\%)=\frac{\left({OD}_{test}-{OD}_{blank}\right)}{\left({OD}_{control}-{OD}_{blank}\right)}\times 100\%$$

#### 3.2.3. Neutralization TNFα Induced Necrosis in L929 Cells

The inhibition of the cytotoxic effect of human TNFα by peptide R1_CC4 and α_CC8 was measured in the L929 cell line. L929 cells were seeded at a density of 8000 cells/well in a 96-well plate and incubated for 24 h in RPMI 1640 with 10% FBS at 37 °C. The experiment was divided into a blank group, a control group, a positive control group, and an experimental group. In the experimental group, serial dilutions of R1_CC4 were in equal volume mixed with 10 ng/mL human TNFα, incubated at 37 °C, for 2 h, which were then added to cells at 100 μL per well and incubated overnight (17 h). Serially diluted α_CC8 was added to cells and incubated at 37 °C, 5% CO_2_ for 2 h initially, and then 50 μL 10 ng/mL human TNFα was added and incubated overnight (17 h). 1 μg/mL Etanercept was used as a positive control. Subsequently, 100 μL 5% CCK-8 was added per well, followed by incubation of 2 h. The control group consisted of normal cultured cells without TNFα stimulation, while the blank control group contained empty wells without cells. At last, cell viability was confirmed by measuring the absorbance of each well at 450 nm. Cell viability was calculated according to Formula (7). Data was analyzed with SPSSAU (https://spssau.com/, Accessed on 14 August 2023) using the paired *t*-test to compare the differences between the experimental and control groups. The significance level for the paired *t*-test was denoted as * *p* < 0.05, ** *p* < 0.01, as indicated in the figure legends [57].

#### 3.2.4. Enzyme-Linked Immunosorbent Assay (ELISA)

The hTNFR1 (100 ng) was coated on a 96-well ELISA plate (BIOFIL, Guangzhou, China) overnight at 4 °C, followed by blocking the plate with 200 μL of 3% skim milk solution in PBST (PBS, 0.05% Tween20) for 2 h at 37 °C. For the α_CC8 test, WP9QY (designed to target TNFR1) was used as the control. The experimental conditions of L16R and WP9QY were identical. Cyclic peptides (α_CC8 and WP9QY) were serially diluted 5-fold from 4 μM to 0.00128 μM in PBS buffer. After incubating with the cyclic peptides for 2 h at room temperature, FITC-tagged mAB (Solarbio, Beijing, China) was diluted at 1:500, and was incubated for an additional 2 h at room temperature. Five washes with PBST were carried out between each incubation to remove nonspecific absorbance. After the final wash, the samples were incubated in the dark with freshly prepared TMB Chromogen Solution (Sangon Biotech, Shanghai, China) for 10 min at room temperature to develop the signals. After adding the stop solution (1 M H_2_SO_4_), the plates were read at 450 nm on a plate reader. The raw data were processed to calculate the K_d_ value.

## 4. Conclusions

In this research, a combination of computational simulations and bioactivity assays was used to design novel cyclic peptides targeting the TNFα-TNFR1 protein–protein interaction, which is known to be a possible cause of inflammation. Firstly, the exact TNFα-TNFR1 interaction mechanism was identified in order to determine the core interaction region and key residues, as well as the truncated sites of initial linear peptides. Residue energy decomposition and alanine mutation results demonstrated that the important residues were mostly uncharged or long-chain-containing benzene rings. For TNFR1, 11 key residues located in CRD2 and CRD3 constitute the TNFα binding site. For TNFα, 10 key residues in two regions constitute the binding site of TNFR1. Based on these, 11 linear peptide sequences were designed, and two cyclization strategies of amide bond and disulfide bond were applied in order to improve the stability of linear peptides. Then, two cyclic peptides, R1_CC4 and α_CC8, originating from TNFR1 and TNFα with high stability and affinity, were screened out by MM-PBSA binding free energy calculations. Further, they were detected to be able to improve cell viability significantly by inhibiting TNFα-mediated necrosis, also showing no cytotoxicity by experiment in vitro. Moreover, α_CC8 exhibits higher biological activity and effective concentration than R1_CC4 and, thus, was selected for further optimization, focusing on enhancing its affinity and solubility by virtual mutations. Finally, L16R with improved water solubility and affinity was obtained based on the virtual mutation of α_CC8 and could be considered as a novel potential cyclic peptide inhibitor to disturb the interaction of TNFα-TNFR1. This study provides a promising computational design strategy for cyclic peptides with better anti-inflammatory effects, and we believe this could bring more choice and hope for RA patients in the future.

## Figures and Tables

**Table 1:** Binding free energy (∆G_bind_) and its components (unit: kcal·mol^−1^).

Complex	ΔE_ele_	ΔE_vdw_	ΔG_PB_	ΔG_nonp_	ΔE_MM_	ΔG_solv_	ΔG_bind_
TNFα-TNFR1	−33.51 ± 0.18	−88.74 ± 0.05	80.73 ± 0.17	−9.28 ± 0.01	−122.25 ± 0.19	71.44 ± 0.17	−50.80 ± 0.06

Note: ∆G_bind_ ≈ ∆H = ΔE_ele_ + ΔE_vdw_ + ΔG_PB_ + ΔG_nonp_, ΔE_MM_ = ΔE_ele_ + ΔE_vdw_, ΔG_solv_ = ΔG_PB_ + ΔG_nonp_.

**Table 2:** Binding energy of different cyclic peptide-TNFα complex. (unit: kcal·mol^−1^).

Region	Sequence	Serial Number	ΔG_bind_ (R1_GPX-TNFα)	ΔG_bind_ (R1_CCX-TNFα)
Region 1	ASENHLRHCL	1	−23.36 ± 0.03	−24.38 ± 0.04
	FTASENH	2	−21.62 ± 0.02	−18.58 ± 0.04
	GSFTASENHLR	3	−37.73 ± 0.03	−20.67 ± 0.04
	ESGSFTASENHLRH	4	−27.08 ± 0.03	−41.13 ± 0.07
Region 2	CRKEMGQV	5	−18.99 ± 0.02	−15.46 ± 0.03
	SKCRKEMGQVEI	6	−23.04 ± 0.02	−11.53 ± 0.05
	SKCRKEMGQVEISS	7	−15.84 ± 0.02	−14.11 ± 0.03
Region 3	HYWSENLFQ	0	−21.05 ± 0.03	-
	WP9QY	0	-	−22.16 ± 0.08

**Table 3:** Binding energy of different CPs-TNFR1 complex (unit: kcal·mol^−1^).

Region	Sequence	Serial Number	ΔG_bind_ (α_GPX-TNFR1)	ΔG_bind_ (α_CCX-TNFR1)
Region 1	TISRIAVSYQTKVNLLS	8	−23.98 ± 0.06	−35.10 ± 0.07
Region 2	THVLLTN	9	−21.05 ± 0.03	−21.02 ± 0.03
	THVLLTI	10	-	−21.97 ± 0.03
	THVLLTNI	11	−5.09 ± 0.01	−17.72 ± 0.08

**Table 4:** Energy distribution of different complexes and the corresponding mutants. (unit: kcal·mol^−1^).

Complex	Mutant	∆E_ele_	∆E_vdw_	∆G_PB_	∆G_nonp_	∆G_bind_
α_CC8-TNFR1	-	−220.28 ± 0.33	−39.82 ± 0.04	230.90 ± 0.29	−5.90 ± 0.01	−35.10 ± 0.07
Mutant-TNFR1	L16R	−162.11 ± 0.22	−45.50 ± 0.04	174.56 ± 0.21	−6.00 ± 0.01	−39.03 ± 0.06
	L16H	−218.54 ± 0.30	−40.05 ± 0.03	231.22 ± 0.27	−5.80 ± 0.01	−33.17 ± 0.05
	L16K	−374.62 ± 0.30	−43.31 ± 0.03	388.43 ± 0.27	−5.50 ± 0.01	−35.00 ± 0.06
	L16D	−168.32 ± 0.36	−34.33 ± 0.03	181.74 ± 0.33	−5.10 ± 0.01	−26.01 ± 0.06
	L16E	−176.26 ± 0.29	−36.65 ± 0.03	191.16 ± 0.26	−5.38 ± 0.01	−27.13 ± 0.05

## Data Availability

The original file and datasets to download are available https://github.com/bigsmartguy/jiang_nan. Accessed on 14 August 2023.

## Supplementary Materials

The following supporting information can be downloaded at: https://www.mdpi.com/article/10.3390/molecules29215147/s1. Figure S1: Reported TNF-α or TNFR1 inhibitors; Figure S2: The dynamic changes of linear peptide (using peptide 2 as an example) and TNFα simulation process; Figure S3: The effects of different concentrations of TNFα on the survival rate and morphological changes of L929 cells after incubation 17 h; Figure S4: (A) The most representative structures of α_CC8-TNFR1 and TNFα-TNFR1 complexes after clustering. (B) TNF-α and superimposed structure and RMSD; Figure S5: Dynamic cross-correlation maps of TNFα-TNFR1 (A) and α_CC8-TNFR1 (B); Figure S6: Principal component analysis and the first principal component motion diagram of TNFα-TNFR1 (A) and α_CC8-TNFR1 (B). (C) Free energy landscape, low-energy conformation and superimposed conformation of TNFα-TNFR1 and α_CC8-TNFR1 complex; Figure S7: RMSD curves of complexes formed by α_CC8 or mutants with TNFR1; Figures S8 and S9: Schematic diagram of the secondary structure of R1_CC4 and α_CC8. Table S1: The Hbond in TNFα-TNFR1 complex; Tables S2 and S3: Residue-Specific Binding Free Energies of TNFα and TNFR1 (unit: kcal/mol); Table S4: The computational alanine for residues within 5 Å of the binding interface; Tables S5 and S6: Information on different peptides derived from TNFR1 and TNFα; Table S7: Binding free energy of α_CC8-TNFR1 complex; Table S8: Residue energy contribution of α_CC8 in α_CC8-TNFR1 complex; Table S9: Hydrogen bond interaction in α_CC8-TNFR1 complex; Table S10: Physicochemical properties and solubility of α_CC8 mutant. Report 1–4: Structural information of α_CC8, R1_CC4, L16R, WP9QY.
