# Supplementary material for: Discovery of Cyclic Peptide Inhibitors Targeted on TNFα-TNFR1 from Computational Design and Bioactivity Verification
Source: Molecules. 2024 Oct 31;29(21):5147. doi: 10.3390/molecules29215147 (PMC11547827; doi:10.3390/molecules29215147)
Supplement: Supplementary file 1 [file molecules-29-05147-s001.zip › molecules-3252377-supplementary.pdf]

# Supporting Information

## Discovery of Cyclic peptide Inhibitors Targeted on TNFα-TNFR1 from Computational Design and Bioactivity Verification

**Jiangnan Zhang[1], Huijian Zhao[1], Qianqian Zhou[1], Xiaoyue Yang[1], Haoran, Qi[1], Yongxing Zhao[1,2,*], Longhua Yang[1,*]**

[1]School of Pharmaceutical Sciences & Key Laboratory of Advanced Drug Preparation Technologies, Ministry of Education, Zhengzhou University, Zhengzhou, China
[2] Henan Key Laboratory of Nanomedicine for Targeting Diagnosis and Treatment, Zhengzhou 450001, China

*** Correspondence:**
Longhua Yang or Yongxing Zhao
yanglh@zzu.edu.cn or zhaoyx@zzu.edu.cn

**Supplementary Tables**

Table S1. The Hbond in TNFα-TNFR1 complex ........................................................................8
Table S2. Residue-Specific Binding Free Energies of TNFα (unit: kcal/mol) .................................7
Table S3. Residue-Specific Binding Free Energies of TNFR1 (unit: kcal/mol)...............................4
Table S4. The computational alanine for residues within 5 Å of the binding interface .................12
Table S5. Information on different peptides derived from TNFR1 ...............................................13
Table S6. Information on different peptides derived from TNFα ..................................................13
Table S7. Binding free energy of α_CC8-TNFR1 complex (unit: kcal/mol) ................................13
Table S8. Residue energy contribution of α_CC8 in α_CC8-TNFR1 complex (unit: kcal/mol) ....14
Table S9. Hydrogen bond interaction in α_CC8-TNFR1 complex ................................................14
Table S10. Physicochemical properties and solubility of α_CC8 mutant ......................................14

**Supplementary Figures**

Figure S1. Reported TNF-α or TNFR1 inhibitors. (A) Small molecules; (B) aptamers ................15
Figure S2. The dynamic changes of linear peptide (using peptide 2 as an example) and TNFα simulation process. Peptide 2 and TNFα were represented by blue and ribbon, respectively.........16
Figure S3. The effects of different concentrations of TNFα on the survival rate and morphological changes of L929 cells after incubation 17 hours (Magnification ×100). .......................................16
Figure S4. (A) The most representative structures of α_CC8 -TNFR1 and TNFα-TNFR1 complexes after clustering. (B) TNF-α and superimposed structure and RMSD. The red and green bands represent TNFα and α_CC8 and their complexes with TNFR1, respectively. ......................17
Figure S5. Dynamic cross-correlation maps of TNFα-TNFR1 (A)and α_CC8-TNFR1 (B). .........18
Figure S6. Principal component analysis and the first principal component motion diagram of TNFα-TNFR1 (A) and α_CC8-TNFR1(B). (C) Free energy landscape, low-energy conformation and superimposed conformation of TNFα-TNFR1 and α_CC8-TNFR1 complex..........................19
Figure S7. RMSD curves of complexes formed by α_CC8 or mutants with TNFR1. ....................19
Figure S8. Schematic diagram of the secondary structure of R1_CC4...........................................20
Figure S9. Schematic diagram of the secondary structure of α_CC8.............................................20

**Supplementary Report**

Report 1 Structural information of α_CC8 ......................................................................................21
(1) detection report...........................................................................................................................21
(2) HPLC result.................................................................................................................................22
(3) MS report.....................................................................................................................................23
Report 2 Structural information of R1_CC4 ....................................................................................24
(1) detection report............................................................................................................................24
(2) HPLC report ................................................................................................................................25
(3) MS report.....................................................................................................................................26
Report 3 Structural information of α_CC8 mutated L16R.............................................................27
(1) detection report............................................................................................................................27
(2) HPLC report ................................................................................................................................28
(3) MS report.....................................................................................................................................29
Report 4 Structural information of WP9QY ...................................................................................30

(1) detection report........................................................................................................30
(2) HPLC report ...............................................................................................................31
(3) MS report...................................................................................................................32

Table S1. The Hbond in TNFα-TNFR1 complex.

| Acceptor | DonorH | Donor | Occupancy (%) | Average distance(Å) |
|---|---|---|---|---|
| GLU_127@OE1 | ARG_68@H | ARG_68@N | 50.69 | 2.86 |
| TRP_107@O | ASN_137@HD22 | ASN_137@ND2 | 37.43 | 2.86 |
| THR_79@OG1 | ASN_110@HD21 | ASN_110@ND2 | 20.29 | 2.91 |
| SER_95@O | ARG_77@HH21 | ARG_77@NH2 | 16.54 | 2.85 |
| TYR_87@OH | ALA_62@H | ALA629@N | 15.93 | 2.91 |
| SER_86@O | HIE_66@H | HIE63@N | 15.16 | 2.92 |
| GLU_127@OE2 | ARG_68@H | ARG_68@N | 15.07 | 2.86 |
| HIE_73@O | GLN_113@HE22 | GLN_113@NE2 | 11.93 | 2.86 |

Note: residues in TNFα were marked in red. residues in TNFR1 were marked in black

Table S1. Residue-Specific Binding Free Energies of TNFα. (unit: kcal·mol$^{-1}$)

| | van der Waals | | | Electrostatic | | | Polar Solvation | | | TOTAL | | |
|---|---|---|---|---|---|---|---|---|---|---|---|---|
| Residue number | Avg. | Std.Dev. | Std.Err. of Mean | Avg. | Std.Dev. | Std.Err. of Mean | Avg. | Std.Dev. | Std.Err. of Mean | Avg. | Std.Dev. | Std.Err. of Mean |
| TYR87 | -5.648 | 0.736 | 0.005 | -2.001 | 0.851 | 0.005 | 4.388 | 0.802 | 0.005 | -3.261 | 0.892 | 0.006 |
| ILE97 | -3.200 | 0.611 | 0.004 | -0.450 | 0.427 | 0.003 | 0.648 | 0.401 | 0.003 | -3.002 | 0.547 | 0.003 |
| SER86 | -4.077 | 0.519 | 0.003 | -2.612 | 0.817 | 0.005 | 4.102 | 0.847 | 0.005 | -2.587 | 1.019 | 0.006 |
| LEU75 | -3.392 | 0.544 | 0.003 | 0.385 | 0.405 | 0.003 | 0.628 | 0.569 | 0.004 | -2.379 | 0.538 | 0.003 |
| ASN137 | -1.483 | 0.723 | 0.005 | -2.176 | 2.143 | 0.014 | 2.127 | 0.879 | 0.006 | -1.533 | 0.984 | 0.006 |
| VAL91 | -1.034 | 0.242 | 0.002 | -0.445 | 0.243 | 0.002 | 0.486 | 0.232 | 0.001 | -1.006 | 0.242 | 0.002 |
| ARG82 | -1.169 | 0.190 | 0.001 | -1.583 | 2.477 | 0.016 | 1.875 | 2.881 | 0.018 | -0.877 | 1.298 | 0.008 |
| VAL74 | -1.131 | 0.528 | 0.003 | -0.796 | 0.414 | 0.003 | 1.160 | 0.403 | 0.003 | -0.767 | 0.600 | 0.004 |
| VAL85 | -0.914 | 0.268 | 0.002 | -1.529 | 0.548 | 0.003 | 1.822 | 0.706 | 0.004 | -0.621 | 0.210 | 0.001 |
| LYS90 | -0.189 | 0.300 | 0.002 | -8.229 | 11.624 | 0.074 | 7.877 | 10.605 | 0.067 | -0.541 | 1.115 | 0.007 |
| HIE78 | -0.308 | 0.106 | 0.001 | -1.191 | 0.370 | 0.002 | 1.016 | 0.368 | 0.002 | -0.484 | 0.330 | 0.002 |
| ALA84 | -0.375 | 0.096 | 0.001 | -0.126 | 0.230 | 0.001 | 0.057 | 0.280 | 0.002 | -0.444 | 0.179 | 0.001 |
| THR79 | -0.849 | 0.353 | 0.002 | -1.472 | 1.105 | 0.007 | 1.906 | 0.667 | 0.004 | -0.414 | 0.756 | 0.005 |
| LEU76 | -0.347 | 0.057 | 0.000 | -0.564 | 0.256 | 0.002 | 0.528 | 0.233 | 0.001 | -0.382 | 0.100 | 0.001 |
| ARG138 | -0.723 | 0.812 | 0.005 | -7.813 | 3.667 | 0.023 | 8.187 | 3.902 | 0.025 | -0.350 | 0.783 | 0.005 |
| THR77 | -1.746 | 0.404 | 0.003 | 1.752 | 0.654 | 0.004 | -0.322 | 0.682 | 0.004 | -0.317 | 0.680 | 0.004 |
| PHE124 | -0.374 | 0.178 | 0.001 | -0.184 | 0.249 | 0.002 | 0.242 | 0.244 | 0.002 | -0.316 | 0.126 | 0.001 |
| LEU126 | -0.302 | 0.057 | 0.000 | 0.529 | 0.171 | 0.001 | -0.537 | 0.237 | 0.001 | -0.310 | 0.145 | 0.001 |
| LEU93 | -0.317 | 0.094 | 0.001 | -0.256 | 0.388 | 0.002 | 0.277 | 0.379 | 0.002 | -0.296 | 0.083 | 0.001 |
| ASN92 | -1.036 | 0.318 | 0.002 | 0.647 | 2.033 | 0.013 | 0.094 | 1.529 | 0.010 | -0.296 | 0.825 | 0.005 |
| THR89 | -0.350 | 0.094 | 0.001 | -0.117 | 0.329 | 0.002 | 0.194 | 0.541 | 0.003 | -0.274 | 0.306 | 0.002 |
| HIE73 | -1.674 | 0.510 | 0.003 | -0.902 | 1.493 | 0.009 | 2.310 | 1.378 | 0.009 | -0.265 | 0.758 | 0.005 |
| GLN88 | -1.236 | 0.586 | 0.004 | -1.062 | 2.318 | 0.015 | 2.076 | 1.831 | 0.012 | -0.222 | 1.073 | 0.007 |
| LEU94 | -0.226 | 0.054 | 0.000 | 1.176 | 0.543 | 0.003 | -1.167 | 0.487 | 0.003 | -0.218 | 0.123 | 0.001 |
| ALA96 | -0.818 | 0.338 | 0.002 | 0.261 | 0.695 | 0.004 | 0.350 | 0.663 | 0.004 | -0.207 | 0.377 | 0.002 |
| LEU55 | -0.143 | 0.054 | 0.000 | -0.161 | 0.122 | 0.001 | 0.158 | 0.118 | 0.001 | -0.146 | 0.052 | 0.000 |

| | | | | | | | | | | | |
|---|---|---|---|---|---|---|---|---|---|---|---|
| ILE80 | -0.104 | 0.017 | 0.000 | -0.280 | 0.244 | 0.002 | 0.244 | 0.224 | 0.001 | -0.140 | 0.047 | 0.000 |
| LYS98 | -0.207 | 0.051 | 0.000 | 1.032 | 2.059 | 0.013 | -0.962 | 2.016 | 0.013 | -0.137 | 0.095 | 0.001 |
| THR72 | -0.256 | 0.309 | 0.002 | -0.629 | 0.527 | 0.003 | 0.754 | 0.601 | 0.004 | -0.131 | 0.182 | 0.001 |
| LYS11 | -0.007 | 0.002 | 0.000 | -6.322 | 1.274 | 0.008 | 6.209 | 1.249 | 0.008 | -0.121 | 0.062 | 0.000 |
| SER99 | -0.103 | 0.024 | 0.000 | -0.123 | 0.227 | 0.001 | 0.111 | 0.221 | 0.001 | -0.115 | 0.035 | 0.000 |
| SER95 | -1.144 | 0.529 | 0.003 | -5.034 | 2.951 | 0.019 | 6.069 | 2.183 | 0.014 | -0.109 | 1.150 | 0.007 |
| SER81 | -0.091 | 0.022 | 0.000 | -0.258 | 0.467 | 0.003 | 0.256 | 0.444 | 0.003 | -0.093 | 0.072 | 0.000 |
| ARG6 | -0.002 | 0.001 | 0.000 | -6.099 | 2.116 | 0.013 | 6.020 | 2.076 | 0.013 | -0.081 | 0.112 | 0.001 |
| VAL123 | -0.065 | 0.020 | 0.000 | -0.182 | 0.213 | 0.001 | 0.170 | 0.207 | 0.001 | -0.077 | 0.024 | 0.000 |
| TYR56 | -0.059 | 0.009 | 0.000 | 0.078 | 0.114 | 0.001 | -0.095 | 0.117 | 0.001 | -0.076 | 0.026 | 0.000 |
| GLY129 | -0.047 | 0.013 | 0.000 | -0.321 | 0.109 | 0.001 | 0.299 | 0.109 | 0.001 | -0.069 | 0.019 | 0.000 |
| ILE83 | -0.089 | 0.010 | 0.000 | 0.067 | 0.153 | 0.001 | -0.047 | 0.152 | 0.001 | -0.069 | 0.029 | 0.000 |
| ILE136 | -0.208 | 0.064 | 0.000 | 0.322 | 0.292 | 0.002 | -0.182 | 0.341 | 0.002 | -0.068 | 0.176 | 0.001 |
| LEU120 | -0.083 | 0.020 | 0.000 | 0.476 | 0.144 | 0.001 | -0.456 | 0.138 | 0.001 | -0.063 | 0.017 | 0.000 |
| VAL50 | -0.044 | 0.006 | 0.000 | -0.169 | 0.100 | 0.001 | 0.152 | 0.098 | 0.001 | -0.062 | 0.018 | 0.000 |
| PRO100 | -0.056 | 0.010 | 0.000 | -0.261 | 0.100 | 0.001 | 0.259 | 0.098 | 0.001 | -0.058 | 0.015 | 0.000 |
| SER52 | -0.072 | 0.024 | 0.000 | -0.267 | 0.235 | 0.001 | 0.281 | 0.231 | 0.001 | -0.058 | 0.036 | 0.000 |
| GLY54 | -0.098 | 0.023 | 0.000 | -0.455 | 0.136 | 0.001 | 0.496 | 0.134 | 0.001 | -0.056 | 0.055 | 0.000 |
| ILE118 | -0.062 | 0.014 | 0.000 | 0.347 | 0.133 | 0.001 | -0.341 | 0.127 | 0.001 | -0.056 | 0.015 | 0.000 |
| PRO106 | -0.078 | 0.033 | 0.000 | 0.691 | 0.461 | 0.003 | -0.669 | 0.440 | 0.003 | -0.055 | 0.036 | 0.000 |
| PRO139 | -0.045 | 0.026 | 0.000 | -0.178 | 0.125 | 0.001 | 0.168 | 0.120 | 0.001 | -0.055 | 0.030 | 0.000 |
| GLU116 | -0.017 | 0.003 | 0.000 | 0.647 | 1.600 | 0.010 | -0.684 | 1.564 | 0.010 | -0.055 | 0.080 | 0.001 |
| GLN102 | -0.058 | 0.031 | 0.000 | 0.081 | 0.421 | 0.003 | -0.077 | 0.415 | 0.003 | -0.053 | 0.031 | 0.000 |
| LYS112 | -0.004 | 0.001 | 0.000 | -3.338 | 1.266 | 0.008 | 3.289 | 1.245 | 0.008 | -0.053 | 0.051 | 0.000 |
| ARG31 | -0.002 | 0.001 | 0.000 | -3.041 | 0.818 | 0.005 | 2.994 | 0.802 | 0.005 | -0.048 | 0.057 | 0.000 |
| TYR141 | -0.053 | 0.032 | 0.000 | -0.123 | 0.097 | 0.001 | 0.129 | 0.101 | 0.001 | -0.047 | 0.029 | 0.000 |
| LYS65 | -0.006 | 0.001 | 0.000 | -2.800 | 0.884 | 0.006 | 2.762 | 0.867 | 0.005 | -0.043 | 0.058 | 0.000 |
| GLN25 | -0.031 | 0.030 | 0.000 | -0.324 | 0.406 | 0.003 | 0.313 | 0.394 | 0.002 | -0.042 | 0.038 | 0.000 |
| PRO70 | -0.041 | 0.021 | 0.000 | 0.046 | 0.237 | 0.001 | -0.044 | 0.232 | 0.001 | -0.039 | 0.023 | 0.000 |
| TYR119 | -0.025 | 0.005 | 0.000 | -0.514 | 0.201 | 0.001 | 0.501 | 0.194 | 0.001 | -0.039 | 0.013 | 0.000 |
| PRO51 | -0.035 | 0.012 | 0.000 | 0.196 | 0.178 | 0.001 | -0.198 | 0.175 | 0.001 | -0.038 | 0.017 | 0.000 |
| PHE64 | -0.044 | 0.007 | 0.000 | -0.115 | 0.062 | 0.000 | 0.121 | 0.061 | 0.000 | -0.037 | 0.011 | 0.000 |
| ARG131 | -0.046 | 0.007 | 0.000 | 0.222 | 1.936 | 0.012 | -0.212 | 1.889 | 0.012 | -0.036 | 0.082 | 0.001 |
| ARG32 | -0.001 | 0.000 | 0.000 | -2.628 | 0.669 | 0.004 | 2.595 | 0.657 | 0.004 | -0.034 | 0.046 | 0.000 |
| SER133 | -0.027 | 0.005 | 0.000 | -0.146 | 0.233 | 0.001 | 0.139 | 0.218 | 0.001 | -0.033 | 0.029 | 0.000 |
| LEU132 | -0.037 | 0.004 | 0.000 | -0.018 | 0.110 | 0.001 | 0.023 | 0.104 | 0.001 | -0.033 | 0.013 | 0.000 |
| ILE58 | -0.031 | 0.005 | 0.000 | -0.058 | 0.091 | 0.001 | 0.058 | 0.087 | 0.001 | -0.032 | 0.012 | 0.000 |
| GLU53 | -0.893 | 0.329 | 0.002 | 5.839 | 2.885 | 0.018 | -4.978 | 3.123 | 0.020 | -0.032 | 0.823 | 0.005 |
| GLY121 | -0.014 | 0.003 | 0.000 | -0.490 | 0.159 | 0.001 | 0.476 | 0.154 | 0.001 | -0.029 | 0.009 | 0.000 |
| THR105 | -0.024 | 0.011 | 0.000 | 0.079 | 0.309 | 0.002 | -0.082 | 0.302 | 0.002 | -0.027 | 0.017 | 0.000 |
| SER60 | -0.010 | 0.001 | 0.000 | -0.315 | 0.099 | 0.001 | 0.299 | 0.094 | 0.001 | -0.026 | 0.012 | 0.000 |
| ARG103 | -0.013 | 0.004 | 0.000 | -1.215 | 1.693 | 0.011 | 1.203 | 1.665 | 0.011 | -0.024 | 0.057 | 0.000 |
| GLN47 | -0.015 | 0.002 | 0.000 | -0.158 | 0.231 | 0.001 | 0.150 | 0.221 | 0.001 | -0.023 | 0.014 | 0.000 |

| | | | | | | | | | | | |
|---|---|---|---|---|---|---|---|---|---|---|---|
| VAL62 | -0.018 | 0.003 | 0.000 | -0.185 | 0.072 | 0.000 | 0.184 | 0.069 | 0.000 | -0.019 | 0.010 | 0.000 |
| ARG44 | -0.010 | 0.003 | 0.000 | -1.469 | 1.600 | 0.010 | 1.461 | 1.566 | 0.010 | -0.019 | 0.066 | 0.000 |
| TRP114 | -0.018 | 0.004 | 0.000 | -0.028 | 0.039 | 0.000 | 0.028 | 0.039 | 0.000 | -0.018 | 0.006 | 0.000 |
| CYX101 | -0.019 | 0.003 | 0.000 | 0.151 | 0.098 | 0.001 | -0.149 | 0.095 | 0.001 | -0.017 | 0.005 | 0.000 |
| SER71 | -0.048 | 0.050 | 0.000 | 0.527 | 0.559 | 0.004 | -0.495 | 0.540 | 0.003 | -0.017 | 0.071 | 0.000 |
| ILE154 | -0.014 | 0.001 | 0.000 | -0.044 | 0.053 | 0.000 | 0.042 | 0.051 | 0.000 | -0.016 | 0.006 | 0.000 |
| TYR151 | -0.007 | 0.001 | 0.000 | -0.146 | 0.070 | 0.000 | 0.136 | 0.067 | 0.000 | -0.016 | 0.010 | 0.000 |
| GLY122 | -0.018 | 0.004 | 0.000 | -0.066 | 0.123 | 0.001 | 0.067 | 0.119 | 0.001 | -0.016 | 0.010 | 0.000 |
| ALA156 | -0.008 | 0.001 | 0.000 | -0.187 | 0.074 | 0.000 | 0.179 | 0.072 | 0.000 | -0.016 | 0.005 | 0.000 |
| VAL49 | -0.017 | 0.002 | 0.000 | 0.216 | 0.062 | 0.000 | -0.215 | 0.060 | 0.000 | -0.015 | 0.008 | 0.000 |
| ALA134 | -0.030 | 0.006 | 0.000 | 0.317 | 0.157 | 0.001 | -0.302 | 0.147 | 0.001 | -0.015 | 0.043 | 0.000 |
| LEU48 | -0.012 | 0.001 | 0.000 | -0.127 | 0.076 | 0.000 | 0.125 | 0.075 | 0.000 | -0.014 | 0.009 | 0.000 |
| PRO117 | -0.011 | 0.002 | 0.000 | -0.180 | 0.112 | 0.001 | 0.177 | 0.110 | 0.001 | -0.013 | 0.007 | 0.000 |
| TRP28 | -0.009 | 0.001 | 0.000 | 0.028 | 0.078 | 0.000 | -0.031 | 0.074 | 0.000 | -0.013 | 0.006 | 0.000 |
| GLN149 | -0.003 | 0.001 | 0.000 | -0.192 | 0.105 | 0.001 | 0.183 | 0.101 | 0.001 | -0.012 | 0.009 | 0.000 |
| PHE152 | -0.014 | 0.001 | 0.000 | 0.096 | 0.066 | 0.000 | -0.092 | 0.063 | 0.000 | -0.011 | 0.006 | 0.000 |
| PRO12 | -0.009 | 0.002 | 0.000 | -0.013 | 0.053 | 0.000 | 0.012 | 0.052 | 0.000 | -0.010 | 0.005 | 0.000 |
| GLY108 | -0.015 | 0.009 | 0.000 | 0.182 | 0.172 | 0.001 | -0.177 | 0.167 | 0.001 | -0.010 | 0.009 | 0.000 |
| GLY153 | -0.004 | 0.001 | 0.000 | -0.131 | 0.057 | 0.000 | 0.125 | 0.055 | 0.000 | -0.010 | 0.006 | 0.000 |
| VAL41 | -0.008 | 0.001 | 0.000 | 0.079 | 0.051 | 0.000 | -0.082 | 0.049 | 0.000 | -0.010 | 0.013 | 0.000 |
| ALA109 | -0.012 | 0.007 | 0.000 | 0.047 | 0.138 | 0.001 | -0.045 | 0.135 | 0.001 | -0.009 | 0.009 | 0.000 |
| ILE155 | -0.008 | 0.001 | 0.000 | -0.042 | 0.059 | 0.000 | 0.041 | 0.058 | 0.000 | -0.009 | 0.005 | 0.000 |
| LEU57 | -0.016 | 0.002 | 0.000 | 0.150 | 0.073 | 0.000 | -0.142 | 0.071 | 0.000 | -0.008 | 0.006 | 0.000 |
| CYX69 | -0.011 | 0.003 | 0.000 | 0.130 | 0.132 | 0.001 | -0.126 | 0.128 | 0.001 | -0.008 | 0.009 | 0.000 |
| LEU142 | -0.016 | 0.003 | 0.000 | 0.129 | 0.058 | 0.000 | -0.121 | 0.055 | 0.000 | -0.007 | 0.011 | 0.000 |
| GLY40 | -0.004 | 0.001 | 0.000 | -0.063 | 0.048 | 0.000 | 0.060 | 0.047 | 0.000 | -0.007 | 0.004 | 0.000 |
| ASN46 | -0.006 | 0.001 | 0.000 | -0.012 | 0.077 | 0.000 | 0.011 | 0.075 | 0.000 | -0.007 | 0.006 | 0.000 |
| LEU26 | -0.012 | 0.002 | 0.000 | 0.022 | 0.067 | 0.000 | -0.016 | 0.065 | 0.000 | -0.007 | 0.010 | 0.000 |
| LEU63 | -0.008 | 0.001 | 0.000 | 0.076 | 0.047 | 0.000 | -0.075 | 0.046 | 0.000 | -0.006 | 0.006 | 0.000 |
| GLY24 | -0.005 | 0.002 | 0.000 | -0.033 | 0.164 | 0.001 | 0.032 | 0.157 | 0.001 | -0.006 | 0.013 | 0.000 |
| VAL16 | -0.003 | 0.000 | 0.000 | -0.052 | 0.034 | 0.000 | 0.049 | 0.033 | 0.000 | -0.006 | 0.003 | 0.000 |
| PHE144 | -0.005 | 0.001 | 0.000 | -0.045 | 0.030 | 0.000 | 0.044 | 0.029 | 0.000 | -0.006 | 0.006 | 0.000 |
| GLN27 | -0.004 | 0.001 | 0.000 | -0.006 | 0.088 | 0.001 | 0.004 | 0.085 | 0.001 | -0.006 | 0.005 | 0.000 |
| GLY66 | -0.003 | 0.001 | 0.000 | -0.138 | 0.042 | 0.000 | 0.136 | 0.041 | 0.000 | -0.005 | 0.002 | 0.000 |
| GLY68 | -0.003 | 0.001 | 0.000 | -0.050 | 0.071 | 0.000 | 0.048 | 0.070 | 0.000 | -0.005 | 0.003 | 0.000 |
| LEU43 | -0.005 | 0.001 | 0.000 | 0.028 | 0.044 | 0.000 | -0.027 | 0.043 | 0.000 | -0.005 | 0.003 | 0.000 |
| TYR115 | -0.006 | 0.001 | 0.000 | -0.070 | 0.069 | 0.000 | 0.072 | 0.067 | 0.000 | -0.004 | 0.006 | 0.000 |
| LEU37 | -0.003 | 0.000 | 0.000 | -0.007 | 0.031 | 0.000 | 0.006 | 0.030 | 0.000 | -0.004 | 0.003 | 0.000 |
| TYR59 | -0.011 | 0.001 | 0.000 | 0.214 | 0.065 | 0.000 | -0.206 | 0.063 | 0.000 | -0.004 | 0.005 | 0.000 |
| PRO113 | -0.003 | 0.001 | 0.000 | -0.065 | 0.057 | 0.000 | 0.064 | 0.056 | 0.000 | -0.004 | 0.002 | 0.000 |
| ALA111 | -0.002 | 0.001 | 0.000 | -0.086 | 0.041 | 0.000 | 0.085 | 0.040 | 0.000 | -0.004 | 0.002 | 0.000 |
| GLN67 | -0.003 | 0.001 | 0.000 | -0.052 | 0.127 | 0.001 | 0.052 | 0.125 | 0.001 | -0.003 | 0.003 | 0.000 |
| LEU36 | -0.002 | 0.000 | 0.000 | -0.056 | 0.029 | 0.000 | 0.055 | 0.028 | 0.000 | -0.003 | 0.004 | 0.000 |

| | | | | | | | | | | | | |
|---|---|---|---|---|---|---|---|---|---|---|---|---|
| ALA14 | -0.003 | 0.000 | 0.000 | 0.007 | 0.032 | 0.000 | -0.007 | 0.032 | 0.000 | -0.003 | 0.003 | 0.000 |
| VAL13 | -0.004 | 0.001 | 0.000 | 0.062 | 0.032 | 0.000 | -0.061 | 0.032 | 0.000 | -0.003 | 0.003 | 0.000 |
| GLY148 | -0.001 | 0.000 | 0.000 | -0.046 | 0.028 | 0.000 | 0.044 | 0.027 | 0.000 | -0.002 | 0.004 | 0.000 |
| SER147 | -0.001 | 0.000 | 0.000 | -0.047 | 0.031 | 0.000 | 0.046 | 0.030 | 0.000 | -0.002 | 0.003 | 0.000 |
| ALA22 | -0.002 | 0.001 | 0.000 | 0.015 | 0.043 | 0.000 | -0.015 | 0.042 | 0.000 | -0.002 | 0.006 | 0.000 |
| ALA18 | -0.002 | 0.000 | 0.000 | 0.002 | 0.045 | 0.000 | -0.001 | 0.044 | 0.000 | -0.002 | 0.006 | 0.000 |
| SER9 | -0.002 | 0.001 | 0.000 | -0.025 | 0.070 | 0.000 | 0.025 | 0.069 | 0.000 | -0.002 | 0.003 | 0.000 |
| ASN39 | -0.005 | 0.001 | 0.000 | 0.094 | 0.088 | 0.001 | -0.091 | 0.086 | 0.001 | -0.002 | 0.003 | 0.000 |
| ASN19 | -0.002 | 0.000 | 0.000 | 0.040 | 0.044 | 0.000 | -0.039 | 0.044 | 0.000 | -0.001 | 0.008 | 0.000 |
| ALA33 | -0.001 | 0.000 | 0.000 | -0.021 | 0.025 | 0.000 | 0.020 | 0.024 | 0.000 | -0.001 | 0.002 | 0.000 |
| HIE15 | -0.003 | 0.000 | 0.000 | 0.078 | 0.043 | 0.000 | -0.076 | 0.042 | 0.000 | -0.001 | 0.004 | 0.000 |
| PRO8 | -0.001 | 0.000 | 0.000 | 0.025 | 0.060 | 0.000 | -0.025 | 0.059 | 0.000 | -0.001 | 0.002 | 0.000 |
| ALA145 | -0.001 | 0.000 | 0.000 | -0.016 | 0.026 | 0.000 | 0.016 | 0.026 | 0.000 | -0.001 | 0.002 | 0.000 |
| ALA38 | -0.002 | 0.000 | 0.000 | -0.039 | 0.037 | 0.000 | 0.040 | 0.036 | 0.000 | -0.001 | 0.006 | 0.000 |
| VAL17 | -0.002 | 0.000 | 0.000 | 0.025 | 0.030 | 0.000 | -0.023 | 0.030 | 0.000 | -0.001 | 0.003 | 0.000 |
| GLN21 | -0.001 | 0.000 | 0.000 | 0.021 | 0.044 | 0.000 | -0.021 | 0.044 | 0.000 | -0.001 | 0.002 | 0.000 |
| LEU29 | -0.002 | 0.000 | 0.000 | -0.006 | 0.036 | 0.000 | 0.007 | 0.035 | 0.000 | -0.001 | 0.003 | 0.000 |
| THR7 | -0.001 | 0.000 | 0.000 | 0.006 | 0.058 | 0.000 | -0.005 | 0.057 | 0.000 | -0.001 | 0.003 | 0.000 |
| PRO20 | -0.001 | 0.000 | 0.000 | 0.045 | 0.025 | 0.000 | -0.044 | 0.025 | 0.000 | 0.000 | 0.002 | 0.000 |
| ASN34 | -0.001 | 0.000 | 0.000 | 0.010 | 0.032 | 0.000 | -0.010 | 0.031 | 0.000 | 0.000 | 0.002 | 0.000 |
| GLN61 | -0.009 | 0.001 | 0.000 | 0.002 | 0.085 | 0.001 | 0.007 | 0.082 | 0.001 | 0.000 | 0.011 | 0.000 |
| ASN30 | -0.002 | 0.000 | 0.000 | 0.053 | 0.052 | 0.000 | -0.051 | 0.051 | 0.000 | 0.000 | 0.004 | 0.000 |
| ALA35 | -0.001 | 0.000 | 0.000 | 0.035 | 0.033 | 0.000 | -0.034 | 0.032 | 0.000 | 0.001 | 0.003 | 0.000 |
| VAL150 | -0.004 | 0.001 | 0.000 | 0.067 | 0.040 | 0.000 | -0.062 | 0.038 | 0.000 | 0.001 | 0.007 | 0.000 |
| ASP45 | -0.006 | 0.001 | 0.000 | 1.139 | 1.574 | 0.010 | -1.123 | 1.539 | 0.010 | 0.011 | 0.067 | 0.000 |
| GLU104 | -0.013 | 0.003 | 0.000 | 2.807 | 1.707 | 0.011 | -2.767 | 1.676 | 0.011 | 0.026 | 0.064 | 0.000 |
| ASP143 | -0.003 | 0.001 | 0.000 | 2.731 | 0.962 | 0.006 | -2.700 | 0.941 | 0.006 | 0.028 | 0.066 | 0.000 |
| GLU146 | -0.002 | 0.000 | 0.000 | 2.362 | 0.802 | 0.005 | -2.330 | 0.785 | 0.005 | 0.030 | 0.057 | 0.000 |
| GLU42 | -0.007 | 0.001 | 0.000 | 2.734 | 1.291 | 0.008 | -2.694 | 1.263 | 0.008 | 0.033 | 0.068 | 0.000 |
| GLU23 | -0.004 | 0.002 | 0.000 | 3.438 | 1.115 | 0.007 | -3.389 | 1.094 | 0.007 | 0.045 | 0.052 | 0.000 |
| ASP140 | -0.020 | 0.010 | 0.000 | 5.013 | 1.647 | 0.010 | -4.947 | 1.612 | 0.010 | 0.045 | 0.078 | 0.000 |
| GLU110 | -0.008 | 0.003 | 0.000 | 5.024 | 1.458 | 0.009 | -4.946 | 1.432 | 0.009 | 0.069 | 0.056 | 0.000 |
| ASP10 | -0.003 | 0.001 | 0.000 | 5.496 | 1.022 | 0.006 | -5.401 | 1.003 | 0.006 | 0.093 | 0.052 | 0.000 |
| LEU157 | -0.020 | 0.003 | 0.000 | 6.729 | 1.413 | 0.009 | -6.608 | 1.385 | 0.009 | 0.101 | 0.066 | 0.000 |
| LYS128 | -0.595 | 0.410 | 0.003 | -10.275 | 5.882 | 0.037 | 11.04 | 6.133 | 0.039 | 0.172 | 1.009 | 0.006 |
| ASP130 | -0.168 | 0.023 | 0.000 | -0.744 | 2.156 | 0.014 | 1.442 | 2.150 | 0.014 | 0.531 | 0.632 | 0.004 |
| GLU135 | -0.497 | 0.277 | 0.002 | 4.573 | 6.336 | 0.040 | -3.534 | 6.450 | 0.041 | 0.543 | 1.401 | 0.009 |
| GLN125 | -2.263 | 0.531 | 0.003 | -0.638 | 1.713 | 0.011 | 3.656 | 2.527 | 0.016 | 0.755 | 1.211 | 0.008 |
| GLU107 | -0.308 | 0.191 | 0.001 | 15.586 | 8.675 | 0.055 | -14.43 | 7.747 | 0.049 | 0.843 | 1.068 | 0.007 |
| GLU127 | -2.546 | 0.570 | 0.004 | -7.306 | 2.688 | 0.017 | 17.64 | 3.694 | 0.023 | 4.793 | 2.619 | 0.017 |

Table S3. Residue-Specific Binding Free Energies of TNFR1. (unit: $kcal \cdot mol^{-1}$).

| Residue number | van der Waals | | | Electrostatic | | | Polar Solvation | | | TOTAL | | |
|---|---|---|---|---|---|---|---|---|---|---|---|---|
| | Avg. | Std.Dev. | Std.Err. of Mean | Avg. | Std.Dev. | Std.Err. of Mean | Avg. | Std.Dev. | Std.Err. of Mean | Avg. | Std.Dev. | Std.Err. of Mean |
| LEU67 | -4.315 | 0.474 | 0.003 | -4.023 | 0.716 | 0.005 | 2.596 | 0.302 | 0.002 | -5.742 | 0.798 | 0.005 |
| ARG68 | -2.887 | 0.722 | 0.005 | -25.505 | 4.779 | 0.030 | 22.857 | 4.260 | 0.027 | -5.535 | 1.367 | 0.009 |
| TRP107 | -6.479 | 0.806 | 0.005 | -3.191 | 1.772 | 0.011 | 5.410 | 1.405 | 0.009 | -4.260 | 1.056 | 0.007 |
| LEU111 | -2.945 | 0.745 | 0.005 | -0.213 | 0.280 | 0.002 | 0.519 | 0.410 | 0.003 | -2.639 | 0.761 | 0.005 |
| LEU71 | -2.079 | 0.388 | 0.002 | 0.161 | 0.202 | 0.001 | 0.046 | 0.179 | 0.001 | -1.872 | 0.364 | 0.002 |
| ASN65 | -1.387 | 0.224 | 0.001 | -1.095 | 0.495 | 0.003 | 1.475 | 0.403 | 0.003 | -1.008 | 0.461 | 0.003 |
| HIE69 | -1.772 | 0.447 | 0.003 | 1.293 | 0.663 | 0.004 | -0.415 | 0.735 | 0.005 | -0.893 | 0.445 | 0.003 |
| ASN110 | -1.974 | 0.616 | 0.004 | -1.007 | 2.070 | 0.013 | 2.151 | 1.578 | 0.010 | -0.830 | 1.147 | 0.007 |
| MET80 | -1.388 | 0.687 | 0.004 | 0.110 | 0.212 | 0.001 | 0.470 | 0.297 | 0.002 | -0.807 | 0.519 | 0.003 |
| SER108 | -1.655 | 0.337 | 0.002 | -1.239 | 1.195 | 0.008 | 2.266 | 1.275 | 0.008 | -0.628 | 0.694 | 0.004 |
| PHE115 | -0.614 | 0.438 | 0.003 | -0.582 | 0.551 | 0.003 | 0.698 | 0.555 | 0.004 | -0.498 | 0.426 | 0.003 |
| HIE105 | -0.391 | 0.088 | 0.001 | 0.124 | 0.751 | 0.005 | -0.162 | 0.693 | 0.004 | -0.429 | 0.158 | 0.001 |
| PRO23 | -0.397 | 0.274 | 0.002 | -2.603 | 2.409 | 0.015 | 2.571 | 2.078 | 0.013 | -0.429 | 0.564 | 0.004 |
| TYR38 | -0.182 | 0.025 | 0.000 | -1.098 | 0.244 | 0.002 | 0.861 | 0.216 | 0.001 | -0.419 | 0.096 | 0.001 |
| ARG146 | -0.041 | 0.024 | 0.000 | -19.759 | 2.469 | 0.016 | 19.404 | 2.421 | 0.015 | -0.396 | 0.086 | 0.001 |
| PHE60 | -0.473 | 0.137 | 0.001 | 0.367 | 0.155 | 0.001 | -0.278 | 0.148 | 0.001 | -0.385 | 0.114 | 0.001 |
| LEU39 | -0.247 | 0.038 | 0.000 | 0.313 | 0.204 | 0.001 | -0.426 | 0.184 | 0.001 | -0.360 | 0.071 | 0.000 |
| PHE112 | -0.212 | 0.046 | 0.000 | -0.148 | 0.144 | 0.001 | 0.062 | 0.173 | 0.001 | -0.298 | 0.139 | 0.001 |
| HIE66 | -3.033 | 0.404 | 0.003 | 0.081 | 0.916 | 0.006 | 2.693 | 0.836 | 0.005 | -0.259 | 0.953 | 0.006 |
| ALA62 | -0.588 | 0.300 | 0.002 | -0.964 | 0.493 | 0.003 | 1.304 | 0.472 | 0.003 | -0.248 | 0.496 | 0.003 |
| SER72 | -0.419 | 0.232 | 0.001 | 0.000 | 0.266 | 0.002 | 0.189 | 0.435 | 0.003 | -0.231 | 0.184 | 0.001 |
| GLN24 | -0.204 | 0.242 | 0.002 | -2.199 | 2.655 | 0.017 | 2.209 | 2.531 | 0.016 | -0.194 | 0.407 | 0.003 |
| TYR20 | -0.237 | 0.043 | 0.000 | -0.271 | 0.318 | 0.002 | 0.315 | 0.301 | 0.002 | -0.193 | 0.049 | 0.000 |
| ASP93 | -0.120 | 0.030 | 0.000 | 4.907 | 2.124 | 0.013 | -4.963 | 2.053 | 0.013 | -0.176 | 0.142 | 0.001 |
| VAL90 | -0.025 | 0.005 | 0.000 | 0.242 | 0.072 | 0.000 | -0.344 | 0.090 | 0.001 | -0.127 | 0.071 | 0.000 |
| THR37 | -0.056 | 0.007 | 0.000 | 0.258 | 0.137 | 0.001 | -0.327 | 0.151 | 0.001 | -0.126 | 0.073 | 0.000 |
| CYX70 | -0.271 | 0.050 | 0.000 | 0.207 | 0.258 | 0.002 | -0.058 | 0.249 | 0.002 | -0.122 | 0.119 | 0.001 |
| LYS75 | -0.092 | 0.036 | 0.000 | -5.484 | 1.642 | 0.010 | 5.458 | 1.614 | 0.010 | -0.119 | 0.083 | 0.001 |
| LYS19 | -0.045 | 0.010 | 0.000 | -5.325 | 1.054 | 0.007 | 5.255 | 1.043 | 0.007 | -0.116 | 0.067 | 0.000 |
| ARG99 | -0.011 | 0.005 | 0.000 | -7.563 | 1.916 | 0.012 | 7.463 | 1.879 | 0.012 | -0.112 | 0.075 | 0.000 |
| GLU79 | -0.699 | 0.649 | 0.004 | 5.732 | 2.244 | 0.014 | -5.144 | 2.256 | 0.014 | -0.111 | 0.576 | 0.004 |
| SER74 | -0.131 | 0.045 | 0.000 | -0.017 | 0.200 | 0.001 | 0.038 | 0.192 | 0.001 | -0.110 | 0.045 | 0.000 |
| CYX73 | -0.096 | 0.033 | 0.000 | 0.211 | 0.116 | 0.001 | -0.208 | 0.113 | 0.001 | -0.093 | 0.033 | 0.000 |
| ARG53 | -0.026 | 0.003 | 0.000 | -6.932 | 1.382 | 0.009 | 6.866 | 1.358 | 0.009 | -0.092 | 0.071 | 0.000 |
| HIE22 | -0.069 | 0.026 | 0.000 | -1.246 | 0.693 | 0.004 | 1.224 | 0.679 | 0.004 | -0.091 | 0.059 | 0.000 |
| LYS132 | -0.020 | 0.004 | 0.000 | -6.805 | 1.721 | 0.011 | 6.734 | 1.691 | 0.011 | -0.091 | 0.075 | 0.000 |
| LYS78 | -0.060 | 0.034 | 0.000 | -5.417 | 1.684 | 0.011 | 5.391 | 1.652 | 0.010 | -0.086 | 0.087 | 0.001 |

| | | | | | | | | | | | |
|---|---|---|---|---|---|---|---|---|---|---|---|
| LYS100 | -0.004 | 0.001 | 0.000 | -6.643 | 1.011 | 0.006 | 6.562 | 0.996 | 0.006 | -0.085 | 0.049 | 0.000 |
| GLN113 | -1.068 | 0.681 | 0.004 | -1.607 | 1.999 | 0.013 | 2.594 | 1.646 | 0.010 | -0.082 | 1.267 | 0.008 |
| GLN17 | -0.129 | 0.129 | 0.001 | 0.335 | 0.608 | 0.004 | -0.285 | 0.568 | 0.004 | -0.079 | 0.179 | 0.001 |
| CYX33 | -0.082 | 0.010 | 0.000 | 0.086 | 0.155 | 0.001 | -0.081 | 0.160 | 0.001 | -0.077 | 0.052 | 0.000 |
| HIE34 | -0.051 | 0.009 | 0.000 | 0.695 | 0.156 | 0.001 | -0.718 | 0.171 | 0.001 | -0.075 | 0.075 | 0.000 |
| ARG92 | -0.051 | 0.013 | 0.000 | -5.180 | 1.185 | 0.007 | 5.157 | 1.157 | 0.007 | -0.073 | 0.077 | 0.000 |
| THR50 | -0.018 | 0.002 | 0.000 | -0.522 | 0.133 | 0.001 | 0.475 | 0.122 | 0.001 | -0.065 | 0.022 | 0.000 |
| CYX114 | -0.062 | 0.037 | 0.000 | 0.020 | 0.196 | 0.001 | -0.023 | 0.189 | 0.001 | -0.064 | 0.037 | 0.000 |
| ILE21 | -0.111 | 0.034 | 0.000 | 0.788 | 0.376 | 0.002 | -0.733 | 0.352 | 0.002 | -0.056 | 0.053 | 0.000 |
| TYR40 | -0.072 | 0.015 | 0.000 | 0.626 | 0.268 | 0.002 | -0.606 | 0.258 | 0.002 | -0.052 | 0.017 | 0.000 |
| TYR103 | -0.048 | 0.014 | 0.000 | 0.128 | 0.254 | 0.002 | -0.131 | 0.244 | 0.002 | -0.051 | 0.024 | 0.000 |
| ASN134 | -0.017 | 0.005 | 0.000 | -1.119 | 0.273 | 0.002 | 1.085 | 0.262 | 0.002 | -0.050 | 0.016 | 0.000 |
| VAL14 | -0.025 | 0.014 | 0.000 | -3.065 | 1.649 | 0.010 | 3.044 | 1.620 | 0.010 | -0.046 | 0.065 | 0.000 |
| THR94 | -0.034 | 0.006 | 0.000 | -0.122 | 0.085 | 0.001 | 0.114 | 0.081 | 0.001 | -0.043 | 0.009 | 0.000 |
| ASN148 | -0.026 | 0.014 | 0.000 | -0.679 | 0.739 | 0.005 | 0.664 | 0.720 | 0.005 | -0.041 | 0.026 | 0.000 |
| TYR106 | -0.267 | 0.053 | 0.000 | 0.082 | 0.716 | 0.005 | 0.144 | 0.824 | 0.005 | -0.041 | 0.149 | 0.001 |
| CYX55 | -0.038 | 0.004 | 0.000 | -0.072 | 0.078 | 0.000 | 0.069 | 0.075 | 0.000 | -0.041 | 0.014 | 0.000 |
| THR89 | -0.015 | 0.002 | 0.000 | 0.060 | 0.078 | 0.000 | -0.085 | 0.087 | 0.001 | -0.040 | 0.026 | 0.000 |
| CYX52 | -0.022 | 0.002 | 0.000 | -0.276 | 0.060 | 0.000 | 0.258 | 0.055 | 0.000 | -0.039 | 0.018 | 0.000 |
| SER118 | -0.008 | 0.004 | 0.000 | -0.722 | 0.242 | 0.002 | 0.691 | 0.229 | 0.001 | -0.039 | 0.017 | 0.000 |
| ARG104 | -0.042 | 0.005 | 0.000 | -6.520 | 1.703 | 0.011 | 6.526 | 1.673 | 0.011 | -0.036 | 0.112 | 0.001 |
| CYX29 | -0.013 | 0.002 | 0.000 | -0.287 | 0.085 | 0.001 | 0.267 | 0.079 | 0.001 | -0.034 | 0.010 | 0.000 |
| GLN102 | -0.017 | 0.008 | 0.000 | -0.555 | 0.333 | 0.002 | 0.539 | 0.324 | 0.002 | -0.033 | 0.017 | 0.000 |
| GLY81 | -0.036 | 0.027 | 0.000 | 0.085 | 0.071 | 0.000 | -0.079 | 0.071 | 0.000 | -0.029 | 0.031 | 0.000 |
| ASN41 | -0.039 | 0.009 | 0.000 | 0.162 | 0.476 | 0.003 | -0.152 | 0.458 | 0.003 | -0.028 | 0.021 | 0.000 |
| SER63 | -1.491 | 0.506 | 0.003 | -0.213 | 1.315 | 0.008 | 1.676 | 1.222 | 0.008 | -0.028 | 1.047 | 0.007 |
| CYX15 | -0.025 | 0.007 | 0.000 | -0.106 | 0.089 | 0.001 | 0.105 | 0.087 | 0.001 | -0.026 | 0.008 | 0.000 |
| ASP91 | -0.048 | 0.010 | 0.000 | 5.817 | 1.163 | 0.007 | -5.794 | 1.133 | 0.007 | -0.024 | 0.088 | 0.001 |
| CYX98 | -0.013 | 0.003 | 0.000 | -0.182 | 0.085 | 0.001 | 0.171 | 0.083 | 0.001 | -0.024 | 0.009 | 0.000 |
| GLY58 | -0.023 | 0.007 | 0.000 | 0.063 | 0.068 | 0.000 | -0.063 | 0.066 | 0.000 | -0.024 | 0.008 | 0.000 |
| GLY97 | -0.009 | 0.002 | 0.000 | -0.057 | 0.054 | 0.000 | 0.043 | 0.053 | 0.000 | -0.024 | 0.010 | 0.000 |
| VAL83 | -0.012 | 0.003 | 0.000 | 0.024 | 0.058 | 0.000 | -0.035 | 0.056 | 0.000 | -0.023 | 0.009 | 0.000 |
| LEU119 | -0.005 | 0.002 | 0.000 | -0.369 | 0.132 | 0.001 | 0.353 | 0.124 | 0.001 | -0.022 | 0.011 | 0.000 |
| CYX30 | -0.023 | 0.003 | 0.000 | -0.047 | 0.101 | 0.001 | 0.049 | 0.092 | 0.001 | -0.021 | 0.012 | 0.000 |
| GLN133 | -0.013 | 0.003 | 0.000 | -0.209 | 0.342 | 0.002 | 0.202 | 0.331 | 0.002 | -0.020 | 0.011 | 0.000 |
| CYX120 | -0.003 | 0.001 | 0.000 | -0.504 | 0.104 | 0.001 | 0.488 | 0.099 | 0.001 | -0.019 | 0.014 | 0.000 |
| CYX96 | -0.026 | 0.004 | 0.000 | -0.073 | 0.055 | 0.000 | 0.079 | 0.054 | 0.000 | -0.019 | 0.008 | 0.000 |
| CYX88 | -0.023 | 0.004 | 0.000 | -0.031 | 0.049 | 0.000 | 0.035 | 0.046 | 0.000 | -0.019 | 0.005 | 0.000 |
| SER59 | -0.055 | 0.012 | 0.000 | -0.008 | 0.092 | 0.001 | 0.046 | 0.089 | 0.001 | -0.017 | 0.031 | 0.000 |
| ASN116 | -0.029 | 0.024 | 0.000 | 0.032 | 0.633 | 0.004 | -0.019 | 0.618 | 0.004 | -0.016 | 0.031 | 0.000 |
| VAL95 | -0.019 | 0.003 | 0.000 | 0.030 | 0.050 | 0.000 | -0.025 | 0.048 | 0.000 | -0.014 | 0.005 | 0.000 |
| GLY18 | -0.023 | 0.007 | 0.000 | 0.178 | 0.094 | 0.001 | -0.169 | 0.091 | 0.001 | -0.013 | 0.010 | 0.000 |
| SER27 | -0.008 | 0.002 | 0.000 | -0.010 | 0.113 | 0.001 | 0.005 | 0.110 | 0.001 | -0.013 | 0.004 | 0.000 |

| | | | | | | | | | | | | |
|---|---|---|---|---|---|---|---|---|---|---|---|---|
| SER57 | -0.013 | 0.005 | 0.000 | 0.076 | 0.086 | 0.001 | -0.076 | 0.084 | 0.001 | -0.012 | 0.006 | 0.000 |
| VAL136 | -0.004 | 0.001 | 0.000 | -0.242 | 0.093 | 0.001 | 0.235 | 0.090 | 0.001 | -0.011 | 0.004 | 0.000 |
| CYX43 | -0.012 | 0.002 | 0.000 | -0.019 | 0.124 | 0.001 | 0.021 | 0.121 | 0.001 | -0.010 | 0.006 | 0.000 |
| CYX76 | -0.042 | 0.012 | 0.000 | -0.066 | 0.080 | 0.001 | 0.097 | 0.079 | 0.001 | -0.010 | 0.020 | 0.000 |
| SER86 | -0.006 | 0.001 | 0.000 | 0.024 | 0.050 | 0.000 | -0.028 | 0.048 | 0.000 | -0.010 | 0.004 | 0.000 |
| ASN26 | -0.076 | 0.069 | 0.000 | -0.589 | 0.989 | 0.006 | 0.655 | 1.003 | 0.006 | -0.010 | 0.156 | 0.001 |
| PHE144 | -0.008 | 0.005 | 0.000 | -0.051 | 0.164 | 0.001 | 0.050 | 0.160 | 0.001 | -0.009 | 0.008 | 0.000 |
| CYX117 | -0.009 | 0.003 | 0.000 | 0.007 | 0.167 | 0.001 | -0.007 | 0.161 | 0.001 | -0.009 | 0.005 | 0.000 |
| PRO16 | -0.018 | 0.005 | 0.000 | 0.338 | 0.123 | 0.001 | -0.329 | 0.117 | 0.001 | -0.008 | 0.015 | 0.000 |
| HIE126 | -0.002 | 0.001 | 0.000 | -0.241 | 0.144 | 0.001 | 0.235 | 0.141 | 0.001 | -0.008 | 0.005 | 0.000 |
| LEU121 | -0.006 | 0.002 | 0.000 | 0.128 | 0.169 | 0.001 | -0.130 | 0.164 | 0.001 | -0.008 | 0.021 | 0.000 |
| CYX129 | -0.004 | 0.001 | 0.000 | -0.208 | 0.051 | 0.000 | 0.205 | 0.050 | 0.000 | -0.008 | 0.003 | 0.000 |
| LEU145 | -0.011 | 0.005 | 0.000 | 0.196 | 0.309 | 0.002 | -0.192 | 0.301 | 0.002 | -0.007 | 0.007 | 0.000 |
| SER87 | -0.007 | 0.001 | 0.000 | -0.010 | 0.050 | 0.000 | 0.010 | 0.049 | 0.000 | -0.007 | 0.003 | 0.000 |
| VAL151 | -0.005 | 0.002 | 0.000 | -0.128 | 0.132 | 0.001 | 0.126 | 0.128 | 0.001 | -0.007 | 0.004 | 0.000 |
| PHE143 | -0.002 | 0.001 | 0.000 | -0.216 | 0.067 | 0.000 | 0.212 | 0.066 | 0.000 | -0.006 | 0.003 | 0.000 |
| VAL125 | -0.002 | 0.001 | 0.000 | -0.177 | 0.047 | 0.000 | 0.173 | 0.046 | 0.000 | -0.006 | 0.002 | 0.000 |
| ASN122 | -0.002 | 0.001 | 0.000 | -0.227 | 0.307 | 0.002 | 0.224 | 0.301 | 0.002 | -0.005 | 0.007 | 0.000 |
| THR138 | -0.002 | 0.001 | 0.000 | -0.084 | 0.132 | 0.001 | 0.081 | 0.129 | 0.001 | -0.005 | 0.006 | 0.000 |
| PRO44 | -0.006 | 0.001 | 0.000 | -0.092 | 0.043 | 0.000 | 0.093 | 0.042 | 0.000 | -0.005 | 0.002 | 0.000 |
| ILE85 | -0.008 | 0.001 | 0.000 | -0.029 | 0.052 | 0.000 | 0.033 | 0.050 | 0.000 | -0.004 | 0.004 | 0.000 |
| CYX150 | -0.003 | 0.001 | 0.000 | 0.023 | 0.149 | 0.001 | -0.023 | 0.146 | 0.001 | -0.004 | 0.005 | 0.000 |
| GLN130 | -0.006 | 0.001 | 0.000 | 0.123 | 0.199 | 0.001 | -0.121 | 0.195 | 0.001 | -0.004 | 0.006 | 0.000 |
| GLY123 | -0.001 | 0.000 | 0.000 | -0.185 | 0.076 | 0.000 | 0.182 | 0.076 | 0.000 | -0.004 | 0.009 | 0.000 |
| PRO46 | -0.003 | 0.001 | 0.000 | 0.004 | 0.057 | 0.000 | -0.004 | 0.057 | 0.000 | -0.003 | 0.002 | 0.000 |
| GLY47 | -0.001 | 0.001 | 0.000 | -0.049 | 0.057 | 0.000 | 0.048 | 0.057 | 0.000 | -0.003 | 0.002 | 0.000 |
| SER153 | -0.002 | 0.001 | 0.000 | -0.041 | 0.246 | 0.002 | 0.040 | 0.240 | 0.002 | -0.002 | 0.007 | 0.000 |
| CYX137 | -0.004 | 0.001 | 0.000 | 0.011 | 0.088 | 0.001 | -0.009 | 0.086 | 0.001 | -0.002 | 0.009 | 0.000 |
| CYX139 | -0.002 | 0.001 | 0.000 | 0.008 | 0.055 | 0.000 | -0.008 | 0.054 | 0.000 | -0.001 | 0.004 | 0.000 |
| ILE28 | -0.013 | 0.003 | 0.000 | 0.069 | 0.156 | 0.001 | -0.057 | 0.149 | 0.001 | -0.001 | 0.012 | 0.000 |
| GLN48 | -0.004 | 0.001 | 0.000 | 0.184 | 0.108 | 0.001 | -0.181 | 0.106 | 0.001 | -0.001 | 0.004 | 0.000 |
| ALA141 | 0.000 | 0.000 | 0.000 | 0.028 | 0.058 | 0.000 | -0.028 | 0.057 | 0.000 | -0.001 | 0.002 | 0.000 |
| GLY45 | -0.002 | 0.001 | 0.000 | 0.185 | 0.043 | 0.000 | -0.182 | 0.042 | 0.000 | 0.000 | 0.003 | 0.000 |
| GLY142 | -0.001 | 0.001 | 0.000 | 0.083 | 0.066 | 0.000 | -0.081 | 0.065 | 0.000 | 0.001 | 0.002 | 0.000 |
| SER128 | -0.002 | 0.000 | 0.000 | 0.094 | 0.106 | 0.001 | -0.092 | 0.104 | 0.001 | 0.001 | 0.003 | 0.000 |
| LEU127 | -0.002 | 0.001 | 0.000 | 0.245 | 0.039 | 0.000 | -0.240 | 0.038 | 0.000 | 0.003 | 0.002 | 0.000 |
| ASN101 | -0.005 | 0.002 | 0.000 | 0.246 | 0.194 | 0.001 | -0.238 | 0.189 | 0.001 | 0.004 | 0.006 | 0.000 |
| THR124 | -0.001 | 0.000 | 0.000 | 0.227 | 0.091 | 0.001 | -0.222 | 0.089 | 0.001 | 0.004 | 0.005 | 0.000 |
| SER152 | -0.002 | 0.001 | 0.000 | 0.310 | 0.256 | 0.002 | -0.303 | 0.250 | 0.002 | 0.006 | 0.006 | 0.000 |
| HIE140 | -0.001 | 0.000 | 0.000 | 0.389 | 0.086 | 0.001 | -0.382 | 0.084 | 0.001 | 0.006 | 0.003 | 0.000 |
| THR135 | -0.006 | 0.001 | 0.000 | 0.400 | 0.134 | 0.001 | -0.385 | 0.128 | 0.001 | 0.009 | 0.008 | 0.000 |
| ASP25 | -0.020 | 0.009 | 0.000 | 2.723 | 1.658 | 0.010 | -2.684 | 1.640 | 0.010 | 0.018 | 0.056 | 0.000 |
| THR31 | -0.021 | 0.003 | 0.000 | 0.202 | 0.116 | 0.001 | -0.162 | 0.105 | 0.001 | 0.019 | 0.022 | 0.000 |

| | | | | | | | | | | | |
|---|---|---|---|---|---|---|---|---|---|---|---|
| GLU56 | -0.027 | 0.010 | 0.000 | 6.192 | 1.441 | 0.009 | -6.144 | 1.404 | 0.009 | 0.022 | 0.081 | 0.001 |
| GLN82 | -0.083 | 0.027 | 0.000 | -0.112 | 0.188 | 0.001 | 0.216 | 0.214 | 0.001 | 0.022 | 0.104 | 0.001 |
| ASP49 | -0.005 | 0.001 | 0.000 | 5.415 | 0.946 | 0.006 | -5.370 | 0.934 | 0.006 | 0.040 | 0.059 | 0.000 |
| GLU84 | -0.012 | 0.002 | 0.000 | 5.508 | 1.292 | 0.008 | -5.455 | 1.269 | 0.008 | 0.042 | 0.069 | 0.000 |
| GLU54 | -0.016 | 0.002 | 0.000 | 6.456 | 1.140 | 0.007 | -6.384 | 1.120 | 0.007 | 0.056 | 0.077 | 0.000 |
| GLU131 | -0.009 | 0.001 | 0.000 | 7.204 | 1.425 | 0.009 | -7.128 | 1.404 | 0.009 | 0.067 | 0.076 | 0.000 |
| LYS35 | -0.093 | 0.020 | 0.000 | -6.093 | 1.256 | 0.008 | 6.257 | 1.229 | 0.008 | 0.072 | 0.167 | 0.001 |
| ARG77 | -1.396 | 0.719 | 0.005 | -9.796 | 4.060 | 0.026 | 11.266 | 3.837 | 0.024 | 0.073 | 0.997 | 0.006 |
| GLY36 | -0.027 | 0.004 | 0.000 | -0.203 | 0.081 | 0.001 | 0.332 | 0.118 | 0.001 | 0.102 | 0.080 | 0.001 |
| LYS32 | -0.359 | 0.121 | 0.001 | -12.355 | 2.945 | 0.019 | 12.823 | 2.944 | 0.019 | 0.110 | 1.140 | 0.007 |
| GLU109 | -0.558 | 0.456 | 0.003 | 1.590 | 10.529 | 0.067 | -0.922 | 10.472 | 0.066 | 0.110 | 1.402 | 0.009 |
| ASP51 | -0.016 | 0.002 | 0.000 | 8.059 | 1.459 | 0.009 | -7.922 | 1.428 | 0.009 | 0.121 | 0.090 | 0.001 |
| SER154 | -0.002 | 0.002 | 0.000 | 10.893 | 2.056 | 0.013 | -10.720 | 2.019 | 0.013 | 0.171 | 0.051 | 0.000 |
| GLU149 | -0.011 | 0.004 | 0.000 | 16.154 | 2.077 | 0.013 | -15.871 | 2.025 | 0.013 | 0.271 | 0.074 | 0.000 |
| ASP42 | -0.072 | 0.019 | 0.000 | 12.196 | 3.316 | 0.021 | -11.820 | 3.208 | 0.020 | 0.305 | 0.162 | 0.001 |
| GLU64 | -0.584 | 0.239 | 0.002 | 6.967 | 2.870 | 0.018 | -5.851 | 3.054 | 0.019 | 0.532 | 0.907 | 0.006 |
| THR61 | -0.694 | 0.215 | 0.001 | -0.990 | 0.331 | 0.002 | 2.275 | 0.495 | 0.003 | 0.591 | 0.519 | 0.003 |
| GLU147 | -0.276 | 0.211 | 0.001 | 31.135 | 8.228 | 0.052 | -29.775 | 7.282 | 0.046 | 1.084 | 0.968 | 0.006 |

Table S2. The computational alanine for residues within 5 Å of the binding interface. (unit: kcal·mol$^{-1}$)

| TNFR1 | | TNFα | |
|---|---|---|---|
| Residue number | ΔΔG | Residue number | ΔΔG |
| PHE60ALA | -0.246 | THR72ALA | -0.119 |
| THR61ALA | -0.015 | HIE73ALA | -0.914 |
| ALA62ALA | 0.000 | VAL74ALA | -0.479 |
| SER63ALA | 0.474 | LEU75ALA | -2.113 |
| GLU64ALA | 0.155 | LEU76ALA | -0.113 |
| ASN65ALA | 0.127 | THR77ALA | -0.706 |
| HIE66ALA | -1.084 | ARG82ALA | -1.459 |
| LEU67ALA | -2.414 | ILE83ALA | -0.059 |
| ARG68ALA | -2.854 | ALA84ALA | 0.000 |
| HIE69ALA | -0.888 | VAL85ALA | -0.257 |
| CYX70ALA | -0.021 | SER86ALA | -2.853 |
| LEU71ALA | -2.090 | TYR87ALA | -4.135 |
| ARG77ALA | -1.443 | GLN88ALA | -0.031 |
| LYS78ALA | -0.088 | THR89ALA | 0.043 |
| GLU79ALA | -0.860 | LYS90ALA | -0.597 |
| TRP107ALA | -6.011 | VAL91ALA | -0.957 |
| SER108ALA | 0.854 | ASN92ALA | -0.297 |
| GLU109ALA | -0.459 | ILE97ALA | -3.338 |
| ASN110ALA | -2.245 | GLU135ALA | 0.924 |
| LEU111ALA | -1.781 | ILE136ALA | -0.100 |
| | | ASN137ALA | -2.703 |
| | | ARG138ALA | -1.071 |

Table S3. Information on different peptides derived from TNFR1.

| TNFR1 | Sequence | Number of residues | Serial Number |
|---|---|---|---|
| region1 | ASENHLRHCL | 10 | **1** |
| | FTASENH | 7 | **2** |
| | GSFTASENHLR | 11 | **3** |
| | ESGSFTASENHLRH | 14 | **4** |
| region2 | CRKEMGQV | 8 | **5** |
| | SKCRKEMGQVEI | 12 | **6** |
| | SKCRKEMGQVEISS | 14 | **7** |
| region3 | HYWSENLFQ | 9 | **0** |
| | WP9QY | 5 | **0** |

Table S4. Information on different peptides derived from TNFα.

| TNFα | sequence | Number of residues | Serial Number |
|---|---|---|---|
| region1 | TISRIAVSYQTKVNLLS | 17 | 8 |
| region2 | THVLLTN | 7 | 9 |
| | THVLLTI | 7 | 10 |
| | THVLLTNI | 8 | 11 |

Table S5. Binding free energy of α_CC8-TNFR1 complex. (unit: kcal·mol$^{-1}$)

| Complex | $\Delta E_{ele}$ | $\Delta E_{vdw}$ | $\Delta G_{PB}$ | $\Delta G_{nonp}$ | $\Delta E_{MM}$ | $\Delta G_{solv}$ | $\Delta G_{bind}$ |
|---|---|---|---|---|---|---|---|
| α_CC8-TNFR1 | -220.28 ±0.33 | -39.82 ±0.04 | 230.90 ±0.29 | -5.90 ±0.01 | -260.10 ±0.34 | 225.00 ±0.29 | -35.10 ±0.07 |

Note: $\Delta G_{bind} \approx \Delta H$, $\Delta H = \Delta E_{ele} + \Delta E_{vdw} + \Delta G_{PB} + \Delta G_{nonp}$, $\Delta E_{MM} = \Delta E_{ele} + \Delta E_{vdw}$, $\Delta G_{solv} = \Delta G_{PB} + \Delta G_{nonp}$

Table S6. Residue energy contribution of α_CC8 in α_CC8-TNFR1 complex. (unit: kcal·mol$^{-1}$)

| Residue | $\Delta\Delta G_{vdw}$ | $\Delta\Delta G_{ele}$ | $\Delta\Delta G_{polar}$ | $\Delta\Delta G_{detal}$ |
|---|---|---|---|---|
| Cys19 | -0.05 ±0.01 | -54.86 ±0.10 | 50.77 ±0.09 | -4.13 ±0.01 |
| Asn15 | -2.85 ±0.01 | -5.66 ±0.01 | 6.11 ±0.01 | -2.40 ±0.01 |
| Val14 | -2.29 ±0.01 | -1.71 ±0.01 | 1.94 ±0.01 | -2.06 ±0.01 |
| Lys13 | -1.30 ±0.01 | -28.20 ±0.07 | 28.11 ±0.06 | -1.40 ±0.01 |
| Thr2 | -2.26 ±0.01 | 0.40 ±0.02 | 0.50 ±0.01 | -1.36 ±0.01 |
| Tyr10 | -2.72 ±0.01 | -0.74 ±0.01 | 2.17 ±0.01 | -1.29 ±0.01 |

Table S7. Hydrogen bond interaction in α_CC8-TNFR1 complex

| H-bond acceptor | DonorH | H-bond donor | Occupancy (%) | Average distance(Å) |
|---|---|---|---|---|
| Cys19@OXT | Arg77@HH12 | Arg77@NH1 | 22.76 | 2.79 |
| Cys19@OXT | Arg77@HH22 | Arg77@NH2 | 16.87 | 2.81 |
| Cys73@O | Asn15@HD22 | Asn15@ND2 | 14.55 | 2.86 |

Note: The residues in α_CC8 were shown in red and the residues in TNFR1 were shown in black

Table S8. Physicochemical properties and solubility of α_CC8 mutant

| Name | Iso-electric point | Net charge at pH 7 | Estimated solubility |
|---|---|---|---|
| α_CC8_wt | 10.65 | 2 | Poor water solubility |
| L16R | 11.47 | 4 | Good water solubility |
| L16H | 10.91 | 3.1 | Poor water solubility |
| L16K | 11.07 | 4 | Good water solubility |
| L16D | 10.42 | 2 | Good water solubility |
| L16E | 10.42 | 2 | Good water solubility |

**A**

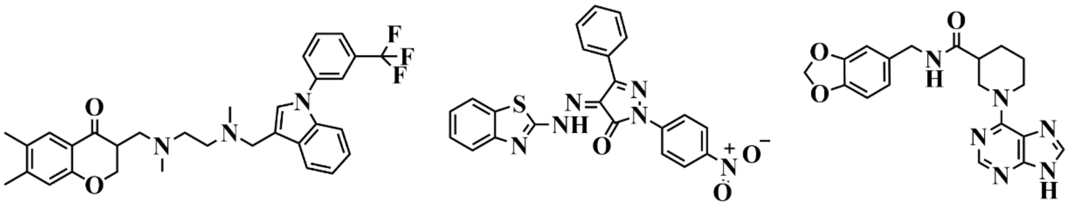

**SPD-304** **T1** **R1**

**B**

| Name | Sequence | Base number |
|---|---|---|
| **VR11** | 5’-TGGTGGATGGCCAGTCGGCGACAAC-3’ | 25 |
| **Apt1-67** | 5'-ACGTCGTATCGAGATAGCACTACTCTTGACCGAAGG GGGACTGCTCGGGATTGCGGATATTCGGTCA-3' | 67 |
| **Apt2-55** | 5'TAGCCAGGGCCTGGGCTAAGTGACCGTGGGCTGGTTGG GTTTGGATTCGGTCACT-3' | 55 |

Figure S1. Reported TNF-α or TNFR1 inhibitors. (A) Small molecules; (B) aptamers

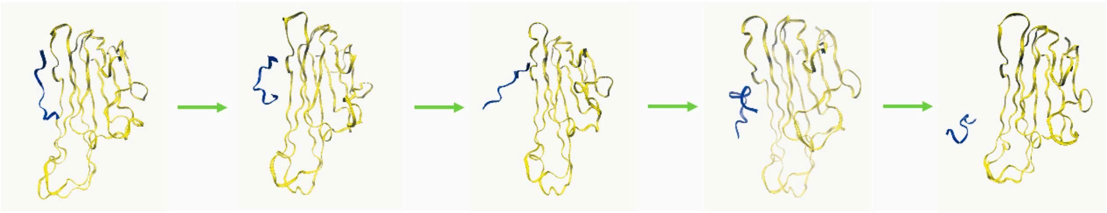

Figure S2. The dynamic changes of linear peptide (using peptide 2 as an example) and TNFα simulation process. Peptide 2 and TNFα were represented by blue and ribbon, respectively.

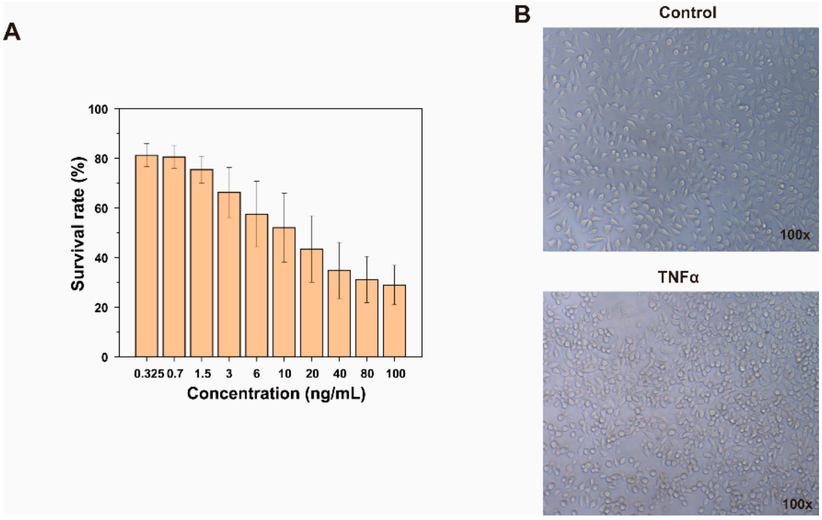


Figure S3. The effects of different concentrations of TNFα on the survival rate and morphological changes of L929 cells after incubation 17 hours (Magnification ×100).

In this study, a 100 ns trajectory from molecular dynamics simulations was subjected to clustering analysis for the non-hydrogen atoms in the α_CC8-TNFR1 and TNFα-TNFR1 complexes. Prior to clustering, water molecules and ions were removed from the trajectory to reduce computational burden. The number of conformations after clustering was set to 10, and distances between frames were calculated based on RMSD after fitting coordinates. The most representative results from the trajectory were selected for analysis.

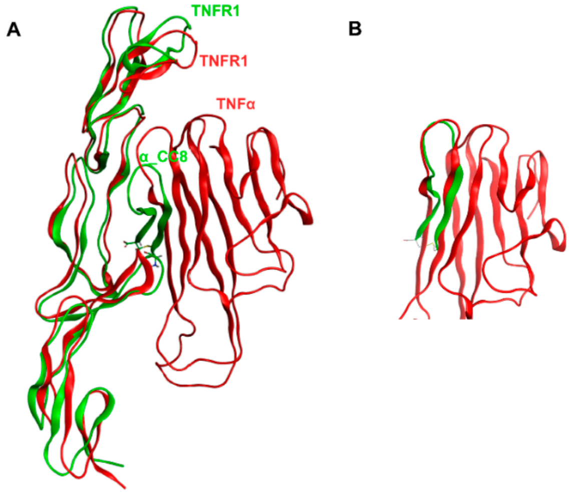


Figure S4. (A) The most representative structures of α_CC8 -TNFR1 and TNFα-TNFR1 complexes after clustering. (B) TNF-α and superimposed structure and RMSD. The red and green bands represent TNFα and α_CC8 and their complexes with TNFR1, respectively.

The Dynamic Cross-correlation Map (DCCM) is a three-dimensional matrix used to analyze differences in dynamic correlations between different systems. It not only provides dynamic correlation information between residues within the time scale of molecular dynamics simulations but also helps identify interactions between residues in biomolecular systems. To better understand the correlation of TNFR1 motion upon binding different ligands in the α_CC8-TNFR1 and TNFα-TNFR1 complexes, we analyzed regions of TNFR1 with significant conformational changes using Origin by computing the dynamic correlation map of C-alpha atoms in the TNFR1 protein.

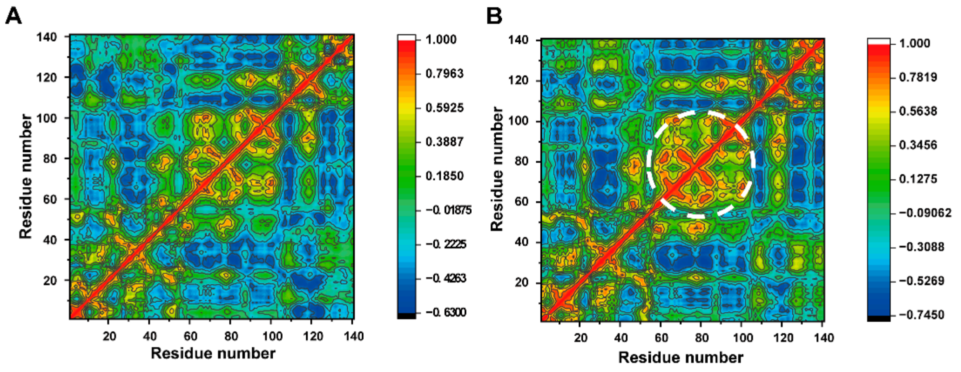


Figure S5. Dynamic cross-correlation maps of TNFα-TNFR1 (A)and α_CC8-TNFR1 (B).

Using all non-hydrogen atoms of the TNFR1 protein from a 100 ns trajectory, principal component analysis (PCA) was conducted.

To reduce computational costs, water molecules were excluded from the trajectory. Subsequently, all atoms except hydrogen atoms were fitted to the first structure of the trajectory using RMSD to generate a reference trajectory average structure. The saved trajectory coordinate information was aligned to the reference structure using RMSD to remove rotational and translational changes, resulting in a processed trajectory for matrix computation. Covariance matrix, eigenvalues, and eigenvectors (i.e., principal components) of coordinates were calculated, with the eigenvector corresponding to the largest eigenvalue selected for projection. Projection of coordinates onto this vector yielded the corresponding principal component information. Additionally, dynamic changes of each principal component were obtained, and visualization and eigenvector plotting were performed using VMD software.

For the analysis of Free Energy Landscape (FEL), typically, two characteristic quantities are utilized. In this study, we employed principal component analysis PC1 and PC2 for plotting. The calculation was carried out using a small program developed by the Lu Tian group (Converting dot distribution to probability distribution, ddtpt). This program converts the scatter distribution obtained from principal components into a probability distribution P(x). It sets the spatial range based on the maximum and minimum data values, and divides the space into small grids according to the user-input grid points in two directions. The probability density at different positions is calculated based on "P(x) = Number of data points in this grid / Total number of data points / Grid area". Subsequently, the probability distribution is transformed into free energy calculation to obtain G(x) according to formulas 2.6 and 2.7. Contour or color maps of the free energy surface are then generated. In this study, scatter distribution plots obtained from PCA were divided into 100 grids along both axes, and the relative binding free energy of each point was outputted. Origin 2023 software was used for data plotting. Finally, the conformations representing the lowest energy were identified based on the calculated free energy values for overlay analysis.

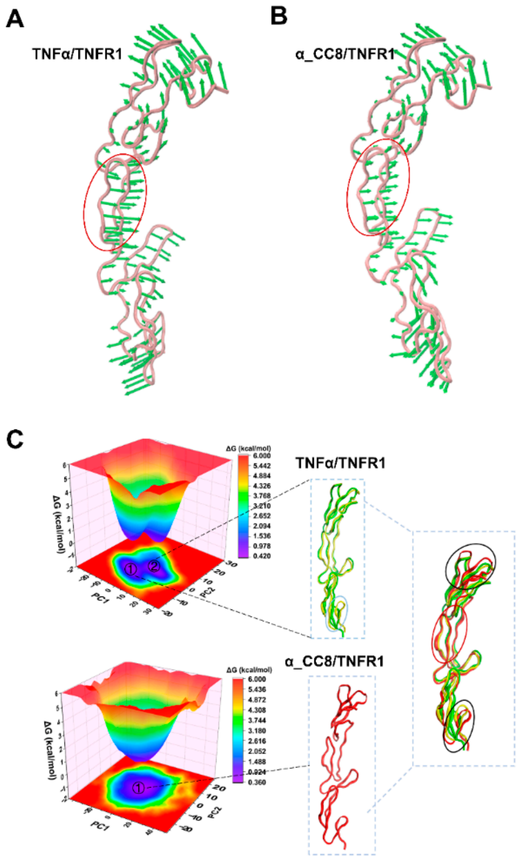


Figure S6. Principal component analysis and the first principal component motion diagram of TNFα-TNFR1 (A) and α_CC8-TNFR1(B). (C) Free energy landscape, low-energy conformation and superimposed conformation of TNFα-TNFR1 and α_CC8-TNFR1 complex.

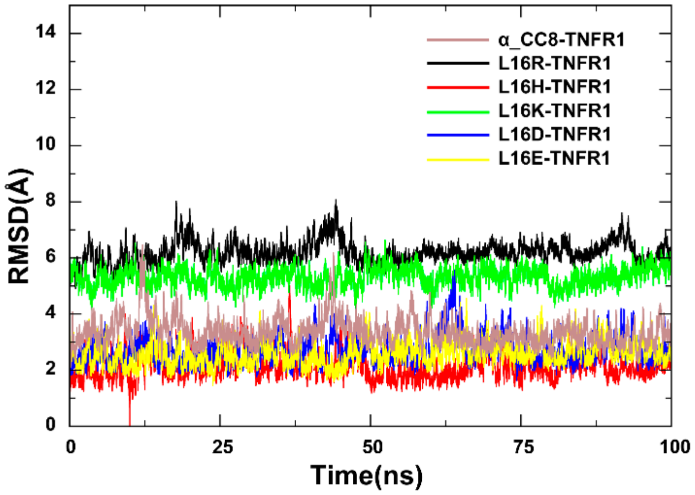


Figure S7. RMSD curves of complexes formed by α_CC8 or mutants with TNFR1.

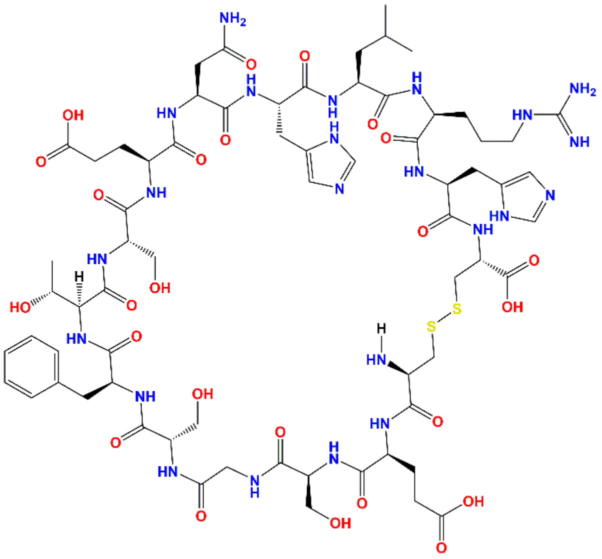

Figure S8. Schematic diagram of the secondary structure of R1_CC4.

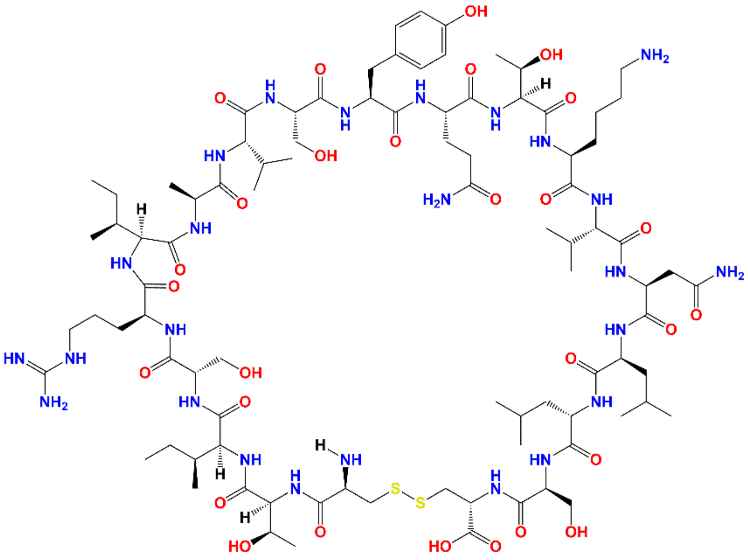

Figure S9. Schematic diagram of the secondary structure of α_CC8.

# Report 1 Structural information of α_CC8

(1) detection report

## CERTIFICATE OF ANALYSIS

| | |
|---|---|
| **Product Name** | S-6591 |
| **Lot No** | DG-101960 |
| **Order ID** | DG#S20220427926 |
| **Sequence** | CTISRIAVSYQTKVNLLSC （Disulfide bond Cys1-Cys19） |
| **Length** | 19AA |
| **Modification** | N/A |
| **Molecular Weight (MW)** | 2097.48 |
| **Storage** | ≤-20℃ |

---

| **Test Items** | **Specifications** | **Results** |
|---|---|---|
| **Purity by HPLC** | >95% | 95.04% |
| **Weight** | 1.0mg*7 | 1.0mg*7 |
| **Peptide Content** | N/A | N/A |
| **Moisture content** | N/A | N/A |
| **Acetic acid content** | N/A | N/A |
| **Appearance** | White to off-white lyophilized powde | Conforms |

**Certified by:Quality Assurance Department**

**Date: 07/08/2022**

**Note: this product is intended for research use only; not for diagnostic or human use.**

**DGpeptides Co., Ltd**

Tel：400-6153-668 http://www.dgpeptides.com

(2) HPLC result

**HPLC**

| | |
|---|---|
| Name | : S-6591 |
| Sequence | : CTISRIAVSYQTKVNLLSC |
| Lot No | : DG-101960 |
| Pump A | : 0. 1% Trifluoroacetic in 100% Water |
| Pump B | : 0. 1% Trifluoroacetic in 100% Acetonrtrile |
| Total Flow | : 1ml/min |
| Wavelength | : 220nm |
| Analytial column type | : SHIMADZU Inertsil ODS-SP (4.6*250mm*5um) |
| Inj. Volume | : 30ul |

| Time | Module | Action | Value |
|---|---|---|---|
| 0.00 | Pumps | B. Conc | 10 |
| 25.00 | Pumps | B. Conc | 70 |
| 25.01 | Pumps | B. Conc | 100 |
| 30.00 | Pumps | B. Conc | 100 |
| 30.01 | Pumps | Stop | |

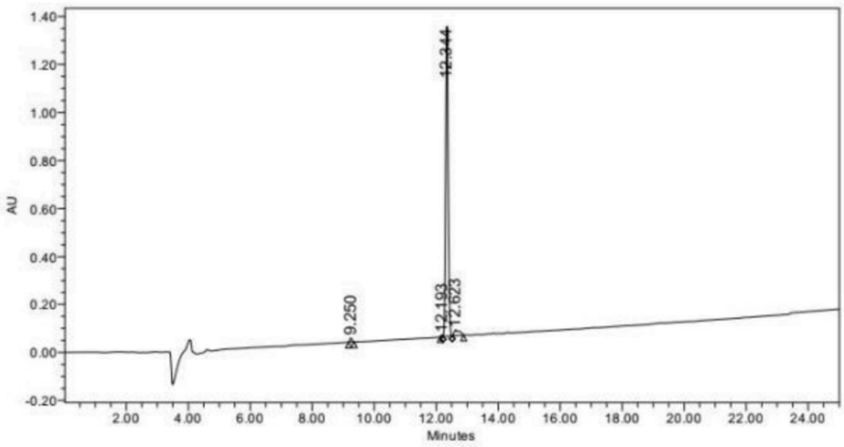


| | RT | Area | % Area | Height |
|---|---|---|---|---|
| 1 | 9.250 | 82893 | 1.07 | 16402 |
| 2 | 12.193 | 20163 | 0.26 | 5791 |
| 3 | 12.344 | 7340102 | 95.04 | 1293091 |
| 4 | 12.623 | 280042 | 3.63 | 23304 |

(3) MS report

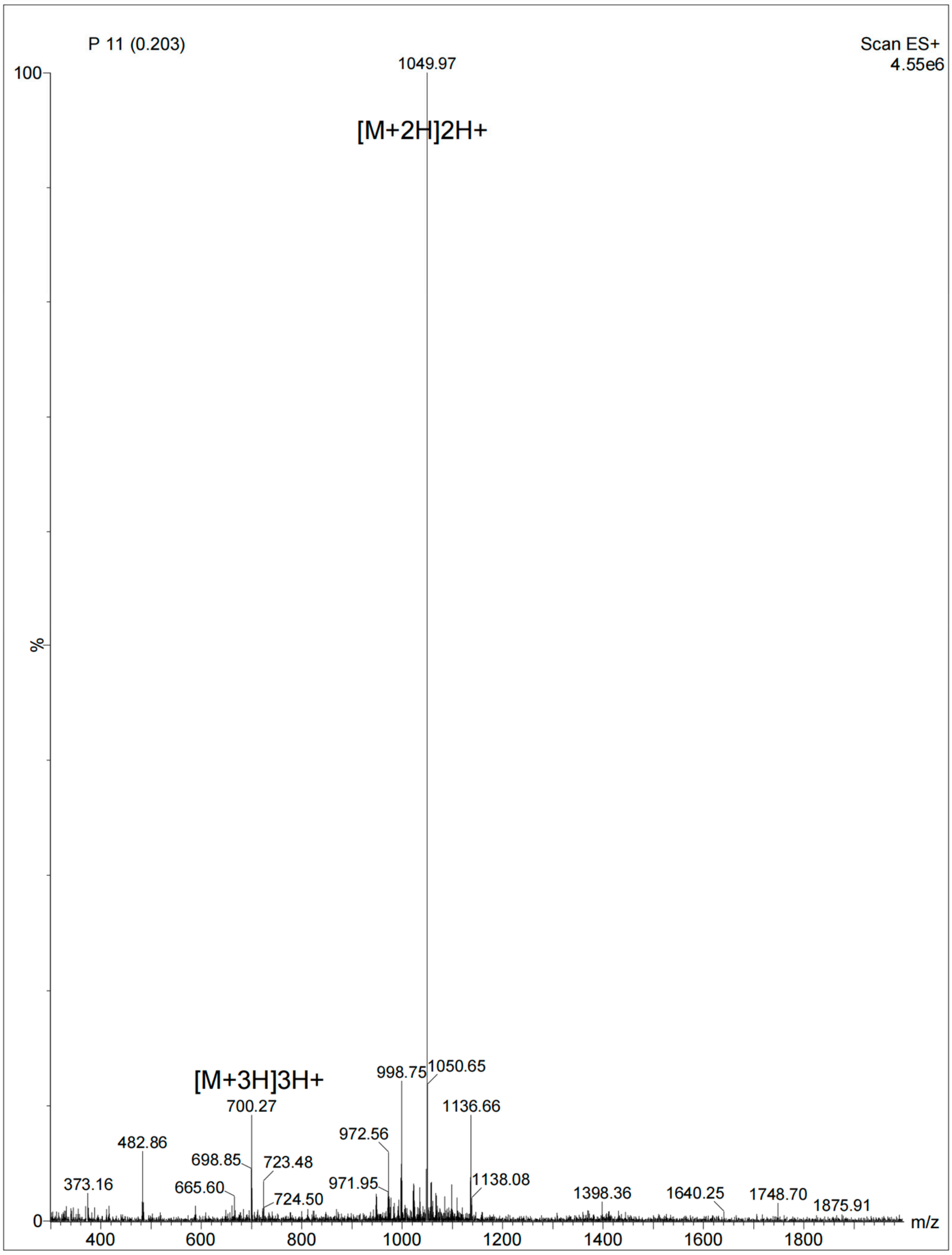

P 11 (0.203)
Scan ES+
4.55e6
1049.97
[M+2H]2H+
100
%
0
[M+3H]3H+
700.27
998.75
1050.65
1136.66
972.56
482.86
698.85
723.48
373.16
665.60
971.95
724.50
1138.08
1398.36
1640.25
1748.70
1875.91
400
600
800
1000
1200
1400
1600
1800
m/z

# Report 2 Structural information of R1_CC4

(1) detection report

## CERTIFICATE OF ANALYSIS

| | |
|---|---|
| **Product Name** | S-6590 |
| **Lot No** | DG-101959 |
| **Order ID** | DG#S20220427926 |
| **Sequence** | CESGSFTASENHLRHC（Disulfide bond Cys1-Cys16） |
| **Length** | 16AA |
| **Modification** | N/A |
| **Molecular Weight (MW)** | 1775.89 |
| **Storage** | ≤-20℃ |

---

| **Test Items** | **Specifications** | **Results** |
|---|---|---|
| **Purity by HPLC** | >95% | 95.72% |
| **Weight** | 1.0mg*7 | 1.0mg*7 |
| **Peptide Content** | N/A | N/A |
| **Moisture content** | N/A | N/A |
| **Acetic acid content** | N/A | N/A |
| **Appearance** | White to off-white lyophilized powde | Conforms |

**Certified by:Quality Assurance Department**

**Date: 06/21/2022**

**Note: this product is intended for research use only; not for diagnostic or human use.**

**DGpeptides Co., Ltd**

**Tel：400-6153-668  http://www.dgpeptides.com**

(2) HPLC report

HPLC

| | |
|---|---|
| Name | : S-6590 |
| Sequence | : CESGSFTASENHLRHC （Disulfide bond Cys1-Cys16） |
| Lot No | : DG-101959 |
| Pump A | : 0.1% Trifluoroacetic in 100% Water |
| Pump B | : 0.1% Trifluoroacetic in 100% Acetonrtrile |
| Total Flow | : 1ml/min |
| Wavelength | : 220nm |
| Analytial column type | : SHIMADZU Inertsil ODS-SP（4.6*250mm*5um） |
| Inj. Volume | : 30ul |

| Time | Module | Action | Value |
|---|---|---|---|
| 0.00 | Pumps | B.Conc | 5 |
| 25.00 | Pumps | B.Conc | 65 |
| 25.01 | Pumps | B.Conc | 100 |
| 30.00 | Pumps | B.Conc | 100 |
| 30.01 | Pumps | Stop | |

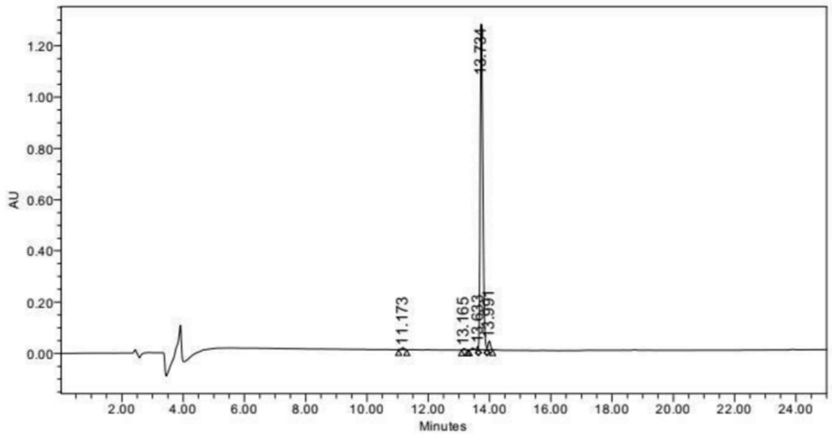


| | RT | Area | % Area | Height |
|---|---|---|---|---|
| 1 | 11.173 | 45617 | 0.58 | 7034 |
| 2 | 13.165 | 25850 | 0.33 | 5205 |
| 3 | 13.633 | 80333 | 1.02 | 13814 |
| 4 | 13.734 | 7571654 | 95.72 | 1263983 |
| 5 | 13.991 | 186575 | 2.36 | 33787 |

(3) MS report

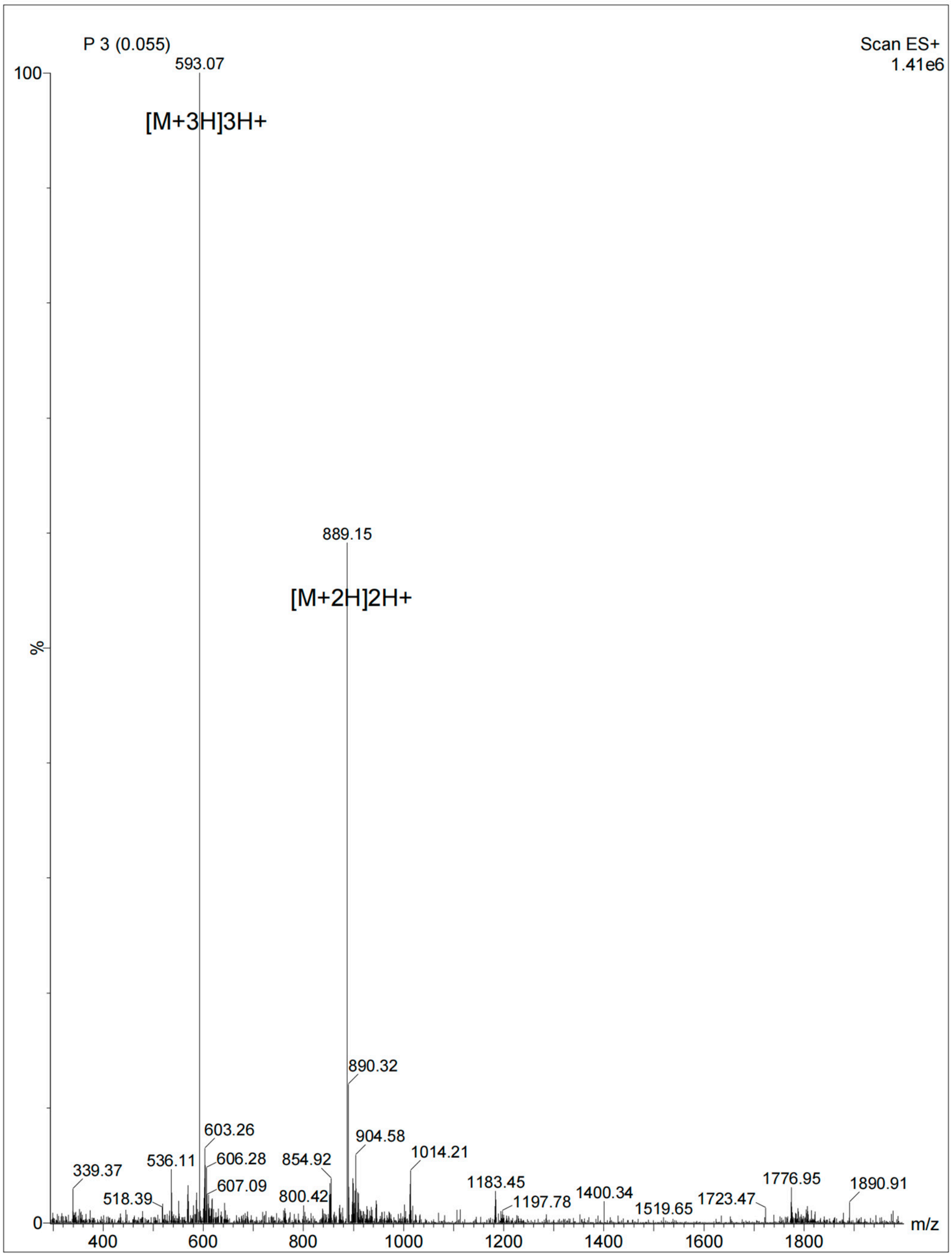

P 3 (0.055)
Scan ES+
1.41e6
100
%
0
593.07
[M+3H]3H+
889.15
[M+2H]2H+
890.32
603.26
606.28
607.09
536.11
518.39
339.37
854.92
800.42
904.58
1014.21
1183.45
1197.78
1400.34
1519.65
1723.47
1776.95
1890.91
400
600
800
1000
1200
1400
1600
1800
m/z

# Report 3 Structural information of α_CC8 mutated L16R

(1) detection report

## CERTIFICATE OF ANALYSIS

| | |
|---|---|
| **Product Name:** | S-8576 |
| **Lot No:** | DG-121137 |
| **Sequence:** | Fitc-CTISRIKVSYQTKVNRLSC (C1-C19) |
| **Dissolution condition:** | 15%ACN+85%H2O |
| **Order ID:** | DG#S202311301184 |
| **Length:** | 19AA |
| **Modification:** | N/A |
| **Molecular Weight (MW) :** | 2700.16 |
| **Storage:** | ≤-20℃ |

| Test Items | Specifications | Results |
|---|---|---|
| **Purity by HPLC** | >95% | 95.10% |
| **Weight** | 3mg(1mg*3) | 3mg(1mg*3) |
| **Peptide Content** | N/A | N/A |
| **Moisture content** | N/A | N/A |
| **Acetic acid content** | N/A | N/A |
| **Appearance** | White to off-white lyophilized powde | Conforms |

**Certified by:Quality Assurance Department**

**Date: 01/04/2024**

**Note: this product is intended for research use only; not for diagnostic or human use.**

(2) HPLC report

**HPLC**

| | |
|---|---|
| Name | : S-8576 |
| Sequence | : Fitc-CTISRIKVSYQTKVNRLSC (C1-C19) |
| Lot No | : DG-121137 |
| Pump A | : 0.1% Trifluoroacetic in 100% Water |
| Pump B | : 0.1% Trifluoroacetic in 100% Acetonrtrile |
| Total Flow | : 1ml/min |
| Wavelength | : 220nm |
| Analytial column type | : SHIMADZU Inertsil ODS-SP（4.6*250mm*5um） |
| Inj. Volume | : 30ul |

| Time | Module | Action | Value |
|---|---|---|---|
| 0.00 | Pumps | B.Conc | 15 |
| 25.00 | Pumps | B.Conc | 75 |
| 25.01 | Pumps | B.Conc | 100 |
| 30.00 | Pumps | B.Conc | 100 |
| 30.01 | Pumps | Stop | |

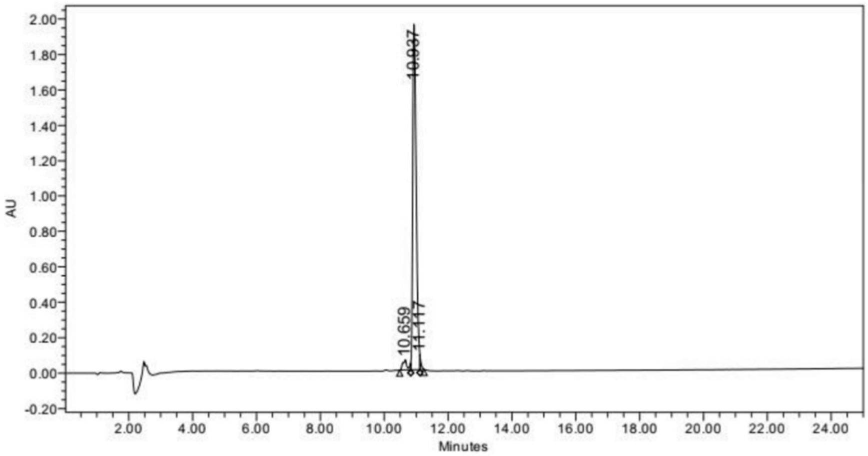


| | RT | Area | % Area | Height |
|---|---|---|---|---|
| 1 | 10.659 | 551756 | 3.61 | 55235 |
| 2 | 10.937 | 14547865 | 95.10 | 1956450 |
| 3 | 11.117 | 197839 | 1.29 | 83833 |

(3) MS report

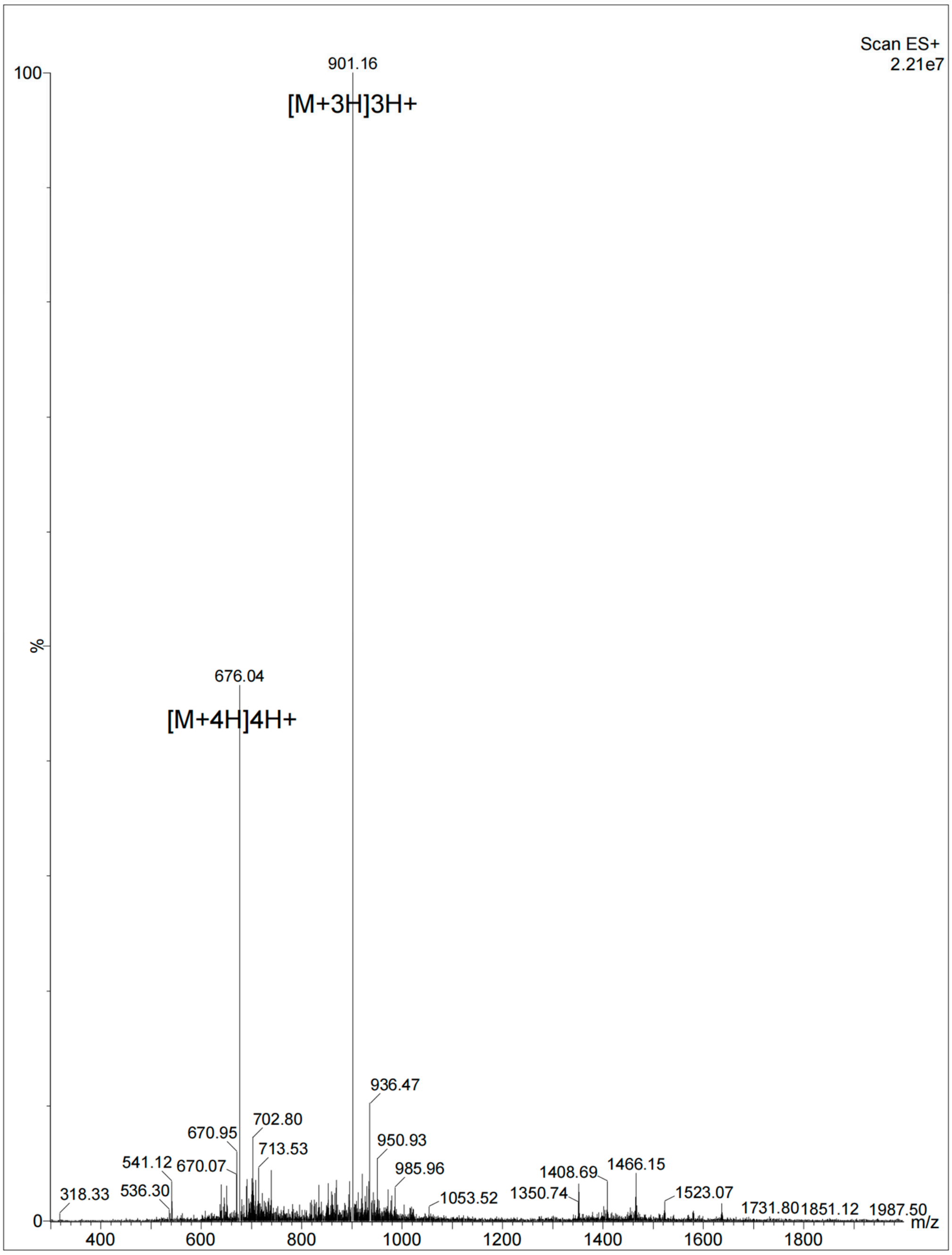
Scan ES+
2.21e7
100
901.16
[M+3H]3H+
%
676.04
[M+4H]4H+
936.47
702.80
670.95
950.93
713.53
541.12
670.07
985.96
1408.69
1466.15
318.33
536.30
1053.52
1350.74
1523.07
1731.80
1851.12
1987.50
0
400
600
800
1000
1200
1400
1600
1800
m/z

# Report 4 Structural information of WP9QY

(1) detection report

## CERTIFICATE OF ANALYSIS

| | |
|---|---|
| **Product Name:** | S-8577 |
| **Lot No:** | DG-121138 |
| **Sequence:** | Fitc-YCWSQYLCY (C2-C8) |
| **Dissolution condition:** | 30%ACN+70%H2O |
| **Order ID:** | DG#S202311301184 |
| **Length:** | 9AA |
| **Modification:** | N/A |
| **Molecular Weight (MW) :** | 1728.95 |
| **Storage:** | ≤-20°C |

| **Test Items** | **Specifications** | **Results** |
|---|---|---|
| **Purity by HPLC** | >95% | 96.05% |
| **Weight** | 3mg(1mg*3) | 3mg(1mg*3) |
| **Peptide Content** | N/A | N/A |
| **Moisture content** | N/A | N/A |
| **Acetic acid content** | N/A | N/A |
| **Appearance** | White to off-white lyophilized powde | Conforms |

**Certified by:Quality Assurance Department**

**Date: 12/15/2023**

**Note: this product is intended for research use only; not for diagnostic or human use.**

(2) HPLC report

**HPLC**

| | |
|---|---|
| Name | : S-8577 |
| Sequence | : Fitc-YCWSQYLCY (C2-C8) |
| Lot No | : DG-121138 |
| Pump A | : 0.1% Trifluoroacetic in 100% Water |
| Pump B | : 0.1% Trifluoroacetic in 100% Acetonrtrile |
| Total Flow | : 1ml/min |
| Wavelength | : 220nm |
| Analytial column type | : SHIMADZU Inertsil ODS-SP（4.6*250mm*5um） |
| Inj. Volume | : 30ul |

| Time | Module | Action | Value |
|---|---|---|---|
| 0.00 | Pumps | B.Conc | 30 |
| 25.00 | Pumps | B.Conc | 90 |
| 25.01 | Pumps | B.Conc | 100 |
| 30.00 | Pumps | B.Conc | 100 |
| 30.01 | Pumps | Stop | |

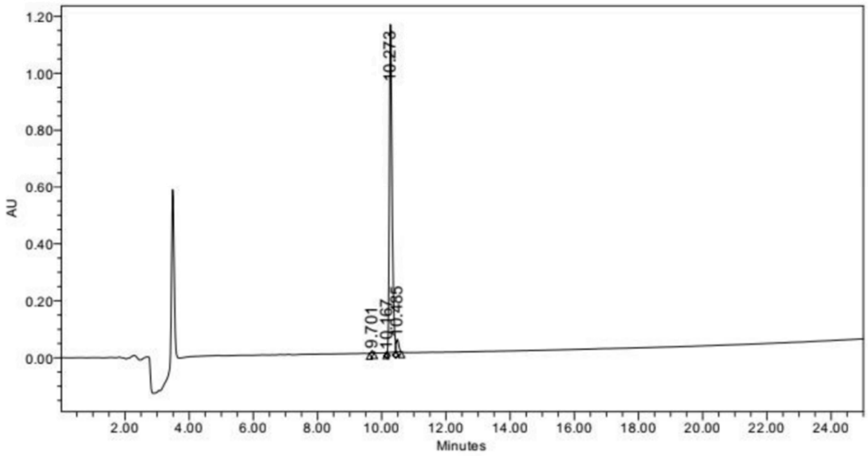


| | RT | Area | % Area | Height |
|---|---|---|---|---|
| 1 | 9.701 | 30651 | 0.43 | 6718 |
| 2 | 10.167 | 533 | 0.01 | 929 |
| 3 | 10.273 | 6828472 | 96.05 | 1152472 |
| 4 | 10.485 | 249865 | 3.51 | 42880 |

(3) MS report

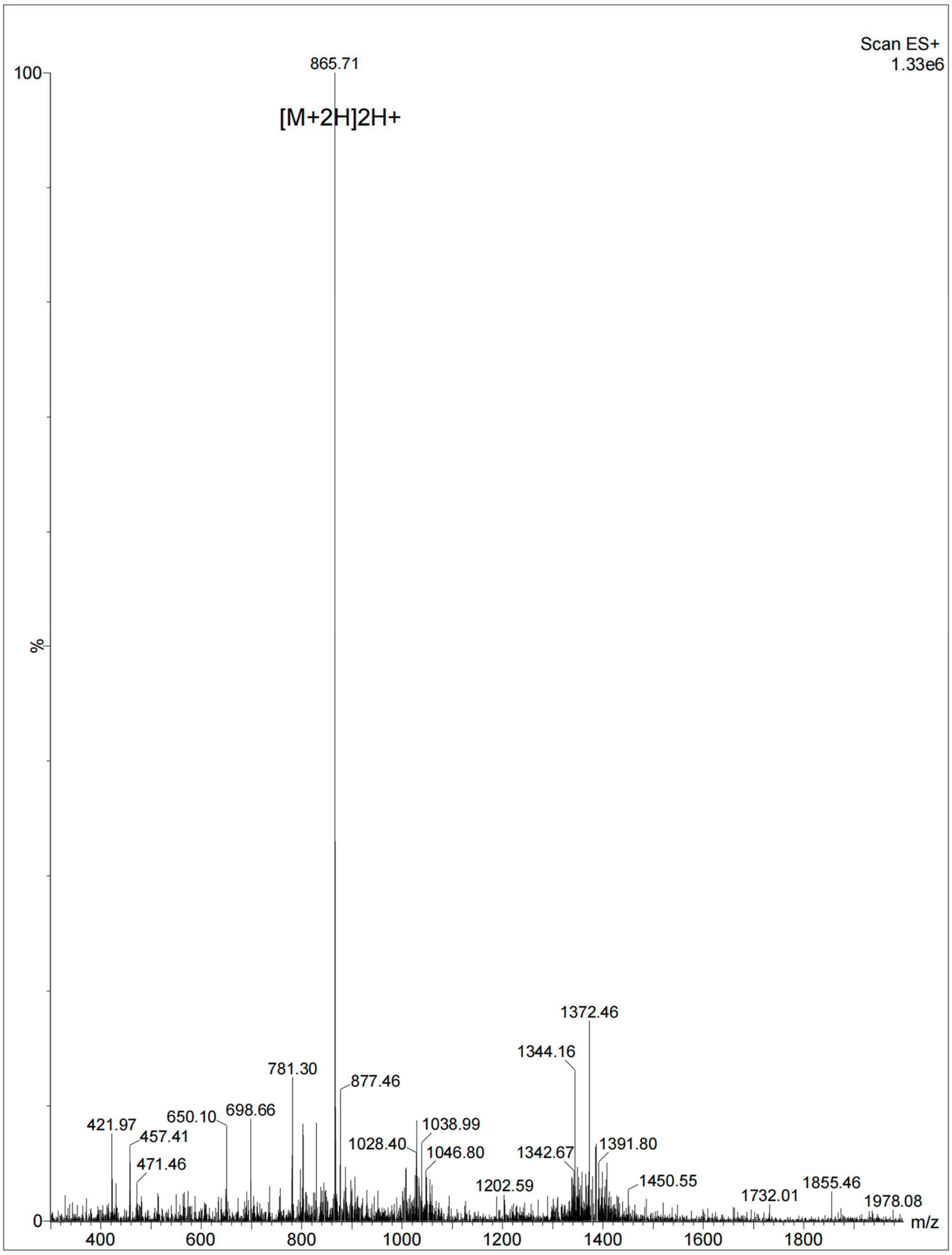

Scan ES+
1.33e6
100
865.71
[M+2H]2H+
%
1372.46
1344.16
781.30
877.46
421.97
650.10
698.66
1038.99
457.41
1028.40
1046.80
1342.67
1391.80
471.46
1202.59
1450.55
1732.01
1855.46
1978.08
0
400
600
800
1000
1200
1400
1600
1800
m/z
